# Amphiphilic Polymer Capped Perovskite Compositing with Nano Zr‐MOF for Nanozyme‐Involved Biomimetic Cascade Catalysis

**DOI:** 10.1002/advs.202304149

**Published:** 2023-08-27

**Authors:** Qiuyu Ye, Enxian Yuan, Jin Shen, Mingli Ye, Qin Xu, Xiaoya Hu, Yun Shu, Huan Pang

**Affiliations:** ^1^ School of Chemistry and Chemical Engineering Yangzhou University Yangzhou 225002 P. R. China

**Keywords:** biomimetic cascade catalysis, biosensing, CsPbX_3_ perovskite nanocrystals, metal–organic frameworks, nanozymes

## Abstract

CsPbX_3_ perovskite nanocrystal (NC) is considered as an excellent optical material and is widely applied in optoelectronics. However, its poor water stability impedes its study in enzyme‐like activity, and further inhibits its application in biomimetic cascade catalysis. Herein, for the first time, the oxidase‐like and ascorbate oxidase‐like activities of an amphiphilic polymer capped CsPbX_3_ are demonstrated, and its catalytic mechanism is further explored. Furthermore, an all‐nanozyme cascade system (multifunctional CsPbBr_3_@Zr‐metal organic framework (Zr‐MOF) and Prussian blue as oxidase‐like and peroxidase‐like nanozyme) is constructed with a portable paper‐based device for realizing the dual‐mode (ratiometric fluorescence and colorimetric) detection of ascorbic acid in a point‐of‐care (POC) fashion. This is the first report on the utilization of all‐inorganic CsPbX_3_ perovskite NC in biomimetic cascade catalysis, which opens a new avenue for POC clinical disease diagnosis.

## Graphical abstract

The oxidase‐like and ascorbate oxidase‐like activities of an amphiphilic polymer capped CsPbX_3_ are demonstrated. Then, an all nanozyme cascade system (multifunctional CsPbBr_3_@Zr‐metal organic framework and Prussian blue as oxidase‐like and peroxidase‐like nanozyme) is constructed with a portable paper‐based device for realizing the dual‐mode (ratiometric fluorescence and colorimetric) neurochemical analysis in a point‐of‐care fashion.

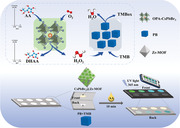

## 1 Introduction

Biocascade catalytic reactors have attracted great attention due to their high efficiency, step‐saving, and good selectivity.^[^ ^1^ ^]^ Despite a wide range of applications in cancer therapy,^[^ ^2^ ^]^ antioxidation,^[^ ^3^ ^]^ and biosensing,^[^ ^4^ ^]^ the inherent drawbacks of natural enzymes used in biocascade catalytic systems, such as harsh catalytic conditions, high preparation and purification costs, and difficulties in recovery and recycling greatly hinder their further application. Compared with natural enzyme, nanozyme has the advantages of simple preparation, low cost, good catalytic stability, and adjustable catalytic activity, and has significantly advanced the development of biocascade catalytic reactors.^[^ ^5^ ^]^ However, the catalytic activity of most nanozymes is unsatisfactory for practical applications, and many approaches for improving nanozymes catalytic activity through various adjustment strategies were introduced, including adjusting size and morphology, vacancy, doping, etc.^[^ ^6^ ^]^ Due to their inherent nanomaterial properties, nanozymes enable efficient cascade catalysis of substrate transformations, and these cascade nanoreactors exhibit increased cascade catalytic stability and efficiency even under complex and harsh physiological conditions.^[^ ^7^ ^]^ Therefore, nanozyme‐engineered biomimetic cascade catalytic nanoreactors showed promising prospects in the field of biosensors and biomedicine.^[^ ^8^ ^]^ Nevertheless, it is still a great challenge to design nanozymes with high activity and stability for catalyzing biocascade reactions.

As a new type of nanomaterial, perovskite nanocrystals (NCs) showed great application prospects in the field of optoelectronics due to its facile synthesis, high fluorescence quantum yield (up to 90%), narrow emission band, and wide coverage of visible light.^[^ ^9^ ^]^ However, there are few reports exploring the nanozyme activity of perovskite NCs for biocatalytic applications. Currently, Li et al.^[^ ^10^ ^]^ reported the peroxidase nanozyme‐like property of CsPbX_3_ NCs using phospholipid membrane encapsulation, and successfully applied it for metabolism analysis and dual‐readout sandwich immunoassay. However, its application in biocascade catalytic reactions has not been reported for several reasons. i) The structural characteristics of perovskite NCs make their stability susceptible to many external perturbations (e.g., polar media, light, oxygen, and heat). Poor water stability, lack of targeting, and potential toxicity caused by leakage of heavy metal ions greatly limit their application in bioassays and biocatalysis. ii) Biocascade catalytic reactions integrate at least two reactions, where the cascade pathways generally follow the sequential manner that the reaction product of one enzyme serves as the substrate for another enzyme, requiring the synergistic effect of multiple enzymes. In contrast, the reported catalytic activity of perovskite nanozymes is relatively rare and limited to peroxidase‐like activity, and the diversity of their nanozymatic activities needs to be exploited.

**Scheme 1:**
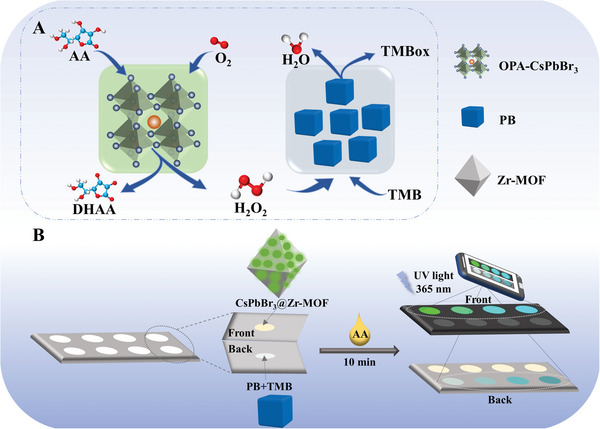
A) The possible reaction pathways of biomimetic cascade catalysis involving OPA‐CsPbBr_3_ NCs and PB. B) Biomimetic cascade nanozyme reactions in a paper‐based device for dual‐mode ratiometric fluorescence and colorimetric detection of AA.

Herein, it is discovered that octylamine‐modified polyacrylic acid (OPA) capped CsPbBr_3_ (OPA‐CsPbBr_3_) NCs have oxidase‐like and ascorbate oxidase‐like activities, while applied it in the biocascade catalytic reactions for dual‐mode neurochemical analysis. First, we prepared CsPbBr_3_ NCs capped by an amphiphilic polymeric of OPA ligand, which showed high stability in the aqueous solution. Importantly, OPA‐CsPbBr_3_ was observed showing oxidase‐like and ascorbate oxidase‐like catalytic activities. The mechanism research was performed by density functional theory (DFT) simulation, which demonstrates that O_2_ was activated by the OPA‐CsPbBr_3_ nanozyme to generate superoxide free radicals (O_2_^•−^) via the oxidase‐like pathway, then the O_2_^•−^ directly oxidizes ascorbic acid (AA) and produces hydrogen peroxide (H_2_O_2_) (**Scheme** **1**A). Metal–organic frameworks (MOFs), constructed by metal (clusters) and organic linkers, possessing large property tunability, high specific surface area, and high porosity,^[^ ^11^ ^]^ have been recognized as a stable matrix to confine nanoparticles which can prevent their migration and aggregation.^[^ ^12^ ^]^ More interestingly, compositing OPA‐CsPbBr_3_ NCs with Zr‐MOF not only improves their fluorescence properties, but also constructs a ratiometric fluorescent probe for AA. By cascading multifunctional CsPbBr_3_@Zr‐MOF with oxidase‐like properties and Prussian blue (PB) with peroxidase‐like properties, an all‐nanozyme cascade system was constructed with a portable paper‐based device for realizing the dual‐mode ratiometric fluorescence and colorimetric detection of AA in a point‐of‐care (POC) fashion (Scheme 1B). This work first enables the application of all‐inorganic CsPbBr_3_ nanozyme in a biomimetic cascade catalytic reaction and its application in POC clinical diagnostic was also demonstrated.

## 2 Results and Discussion

### 2.1 Nanozyme Activity Study

**Figure 1:**
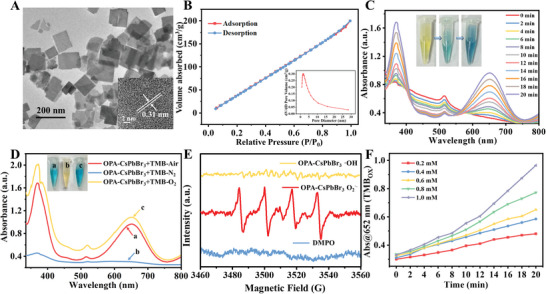
A) TEM and HRTEM images of OPA‐CsPbBr_3_ NCs. B) The time‐dependent N_2_ adsorption–desorption isotherms and inset is the corresponding pore size distribution curve of OPA‐CsPbBr_3_ NCs. C) Real‐time UV–vis absorption spectra of the catalyzing oxidation of TMB by OPA‐CsPbBr_3_ nanozyme, the inset is the corresponding color change of the solution. D) The UV–vis absorption spectra of TMB after catalyzing oxidized by the OPA‐CsPbBr_3_ nanozyme in the air, N_2_, and O_2_ environment. Inset images were the color changes of solutions (a: Air, b: N_2_, and c: O_2_ environment). E) EPR spectra of ^·^OH and O_2_^•−^ in OPA‐CsPbBr_3_ catalytic reaction system. F) Time‐dependent UV–vis absorbance of TMBox at 652 nm produced by OPA‐CsPbBr_3_ catalyzing different concentrations of TMB.

Highly luminescent and stable CsPbBr_3_ NCs were synthesized by an amphiphilic polymer ligand‐assisted reprecipitation method according to our previous work.^[^ ^13^ ^]^ The CsPbBr_3_ NCs with OPA as a capping ligand showed a sharp fluorescent emission peak at 520 nm (Figure S1A, Supporting Information). Transmission electron microscopy (TEM) image of OPA‐CsPbBr_3_ NCs showed that it presented regular nanocube‐shape with relatively transparent flakes, and high‐resolution transmission electron microscopy (HRTEM) showed an interplanar spacing of 0.31 nm, which corresponded to (111) plane of cubic‐phase CsPbBr_3_ NCs (**Figure** **1**A). X‐ray diffraction (XRD) characterization indicated the crystal structure of OPA‐CsPbBr_3_ NCs belonged to the cubic crystal structure (JCPDS 54‐0752) (Figure S1B, Supporting Information).^[^ ^13^ ^b]^ The surface functional groups and elemental states of OPA‐CsPbBr_3_ NCs were further characterized by Fourier transform infrared (FT‐IR) and X‐ray photoelectron spectroscopy (XPS) analysis. As shown in Figure S1C (Supporting Information), the sharp peak between 3500 and 3000 cm^−1^ belonged to the stretching vibration peak of N─H in the amino group, the peak at 1640 cm^−1^ was attributed to the stretching vibration of C═O in the amide bonds, which proved that amphiphilic polymer OPA ligand was successfully bonded to the surface of CsPbBr_3_ NCs. The high‐resolution XPS spectra of C, N, O, Cs, Pb, and Br were displayed in Figures S1D and S2 (Supporting Information). It further verified the successful binding of OPA and oleylamine (OAm) ligands with CsPbBr_3_ NCs. In addition, the specific surface area and surface adsorption of the OPA‐CsPbBr_3_ were studied by Brunauer–Emmett–Teller (BET) analysis. The nitrogen adsorption–desorption and pore size distribution curves showed that OPA‐CsPbBr_3_ NCs had a large specific surface area (337.24 m^2^ g^−1^) and abundant micropores and mesopores (Figure 1B and Table S1, Supporting Information). The high specific surface area and hierarchical pore structure were favorable for the fast conduction of electrons,^[^ ^14^ ^]^ which determines the good potential of OPA‐CsPbBr_3_ NCs in redox reactions.

It could be further found that the fluorescence intensity of OPA‐CsPbBr_3_ could still maintain 80.7% of the initial intensity in water after 10 days (Figure S3, Supporting Information), demonstrating the high water‐stability of the NCs. Based on the large specific surface area, porous structure, and high water‐stability of OPA‐CsPbBr_3_ NCs, its enzymatic‐like activity in aqueous solution was further explored. The oxidation of 3, 3′, 5, 5′‐tetramethylbenzidine (TMB) was used to explore the oxidase‐like and peroxidase‐like activities.^[^ ^8^, ^15^ ^]^ As shown in Figure 1C, when OPA‐CsPbBr_3_ nanocrystal was added to TMB solution, the color of the solution gradually changed from yellow to blue, which means TMBox was produced in the solution. With time increasing, the absorption peak of 370 and 652 nm attributed to TMBox gradually enhanced, and finally the solution color turned to dark blue, suggesting the amount of TMBox increased with time increasing. Overall, it indicates that the OPA‐CsPbBr_3_ NCs possess oxidase‐like catalytic activity.

In order to investigate the mechanism of TMB oxidation catalyzed by OPA‐CsPbBr_3_ NCs, perovskite was added to TMB solution saturated with air, N_2_, and O_2_, respectively (Figure 1D). The peak intensity of TMBox showed stronger in solution saturated with O_2_ and air. However, in the O_2_‐free solution, the color of TMB solution did not turn blue, and the absorption peak of TMBox was very weak and could hardly be monitored. The results confirmed the key role of dissolved oxygen in the OPA‐CsPbBr_3_‐catalyzed TMB system. The presence of reactive oxygen species (ROS) in the catalytic process of OPA‐CsPbBr_3_ was monitored by electron paramagnetic resonance (EPR) analysis. Figure 1E shows four peaks appeared with an intensity ratio of 1:1:1:1, indicating that the ROS of O_2_^•−^ was presented in the catalytic reaction system. The process for the catalytic oxidation of TMB to TMBox by the OPA‐CsPbBr_3_ NCs may be illustrated as follows. O_2_ was catalyzed by OPA‐CsPbBr_3_ NCs to produce O_2_^•−^ at first, and then colorless TMB was catalyzed by O_2_^•−^ to produce blue TMBox (Equations 1 and 2).

(1) $${\mathrm{CsPbBr}}_{3}+O_{2}\to 0_{2}^{\cdot -}$$

(2) $$O_{2}^{\cdot -}+\mathrm{TMB}\to \mathrm{TMBox}$$

Furthermore, the peroxidase‐like activity of OPA‐CsPbBr_3_ NCs was further studied. When OPA‐CsPbBr_3_ NCs were added to the TMB and H_2_O_2_ buffer solution under O_2_‐free conditions, the absorption peak of 370 and 652 nm attributed to TMBox was very weak and almost did not increase over time (Figure S4A, Supporting Information). It indicated that the ability of OPA‐CsPbBr_3_ NCs for catalyzing oxidation of TMB by H_2_O_2_ was very weak, suggesting OPA‐CsPbBr_3_ did not have peroxidase‐like activity. Furthermore, when OPA‐CsPbBr_3_ NCs were added to the TMB and H_2_O_2_ buffer saturated with air, the absorption peak intensity of TMBox enhanced with time increasing (Figure S4B, Supporting Information), it was attributed to the oxidase‐like catalytic activity of OPA‐CsPbBr_3_ NCs. The variation of absorbance with time increasing in the absence and presence of H_2_O_2_ was compared (Figure S4C, Supporting Information). After H_2_O_2_ was added to the reaction system, it did not show a better oxidation effect. In addition, the EPR analysis demonstrated that the peak of ^·^OH was not observed after the addition of H_2_O_2_ to OPA‐CsPbBr_3_ NCs (Figure 1E), which further proved that OPA‐CsPbBr_3_ NCs did not have peroxidase‐like activity.

In addition, the oxidase‐like catalytic activity of the OPA‐CsPbBr_3_ NCs was further evaluated, and steady‐state catalytic kinetics experiments were performed by varying the concentration of TMB (Figure 1F; Figures S5 and S6, Supporting Information). Two steady‐state kinetics parameters, including Michaelis–Menten constant (*K*_m_) and maximum initial velocity (*V*_max_) were calculated according to the Lineweaver–Burk equation (Equations 3 and 4) to validate the catalytic ability.

(3) $$V=\frac{V_{\max}\left[S\right]}{K_{m}+\left[S\right]}$$

(4) $$\frac{1}{V}=\frac{K_{m}}{V_{\max}\left[S\right]}+\frac{1}{V_{\max}}$$

Based on the above equation, the *K*_m_ and *V*_max_ value of OPA‐CsPbBr_3_ NCs was calculated to be 0.095 mm and 4.58 × 10^−7^ m·s^−1^ for TMB, respectively. In Table S2 (Supporting Information), the catalytic ability of OPA‐CsPbBr_3_ NCs was compared with other common nanozymes and nature enzymes with oxidase‐like activity, and it was found that OPA‐CsPbBr_3_ NCs showed a lower *K*_m_ value and larger *V*_max_ value than that of most nanozymes. The results indicated that OPA‐CsPbBr_3_ nanozyme showed strong affinity and high oxidase‐like catalytic performance for the substrate.

### 2.2 Catalyzing the Oxidation of AA and the Mechanism Study

**Figure 2:**
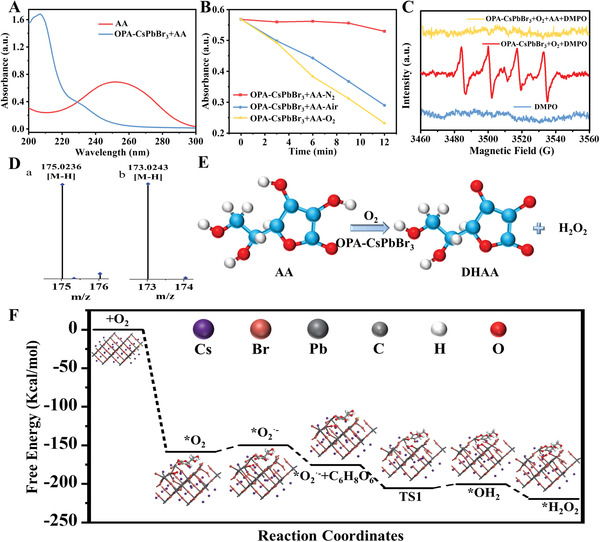
A) UV–vis absorption spectra of AA before and after catalyzed by OPA‐CsPbBr_3_ NCs. B) Time‐dependent UV–vis absorbance for AA at 259 nm catalyzed by OPA‐CsPbBr_3_ under the N_2_‐saturated, Air‐saturated, and O_2_‐saturated environment. C) EPR spectra of O_2_^•−^ in different reaction systems. D) ESI‐MS spectra of a) AA and b) DHAA. E) Reaction pathway of AA oxidation catalyzed by OPA‐CsPbBr_3_ NCs. F) Free energy diagrams of OPA‐CsPbBr_3_ oxidase‐like reactions and the optimized adsorption configurations for each reaction step catalyzing AA.

Based on the oxidase‐like catalytic properties of the OPA‐CsPbBr_3_ nanozyme, its potential to catalyze the oxidation of AA was explored. By monitoring the UV–vis spectra of AA before and after being catalyzed by OPA‐CsPbBr_3_ nanozyme, it was found that the absorption peak of AA at 259 nm disappeared when OPA‐CsPbBr_3_ was added (**Figure** **2**A). It is proved that OPA‐CsPbBr_3_ has special ascorbate oxidase‐like and oxidase‐like properties that can catalyze the oxidation of AA in aqueous solution. In order to study the reaction mechanism of AA catalyzed by OPA‐CsPbBr_3_ nanozyme, the UV–vis spectra of AA solution saturated with Air, N_2_, and O_2_ after the addition of perovskite for different periods was recorded, respectively. (Figure S7, Supporting Information). As shown in Figure 2B, the absorption peak intensity of AA at 259 nm decreased obviously when the solution was saturated with air and O_2_, suggesting the oxidation of AA catalyzed by OPA‐CsPbBr_3_ NCs. Meanwhile, the absorption peak intensity of AA remained stable when the solution was saturated with N_2_. Overall, it proves the participation of O_2_ in the reaction process of AA oxidation. In addition, EPR free radical capture experiment was carried out for further investigation of the oxidation process of AA. As shown in Figure 2C, the formation of O_2_^•−^ during the OPA‐CsPbBr_3_ catalytic reaction was measured by EPR analysis, it may be that O_2_ was catalyzed by OPA‐CsPbBr_3_ to generate O_2_^•−^. When AA was added to the above reaction system, the peak of O_2_^•−^ disappeared, which may be due to the fact that AA, as an efficient electron donor, consumed the O_2_^•−^ produced by the catalytic process, converted into dehydroascorbic acid (DHAA) and produced H_2_O_2_.^[^ ^8^, ^16^ ^]^ Subsequently, the two products of the catalytic reaction were verified, and the oxidation products of AA were analyzed by electrospray ionization mass spectrometry (ESI‐MS) (Figure 2D). After oxidation, the fragment of AA moved down from 175.0236 to 173.0243, indicating that the two ─OH fractions in AA were dehydrogenated and AA was oxidized to DHAA (C_6_H_6_O_6_). The catalytic reaction process was described in Figure 2E. The byproduct H_2_O_2_ was further detected using the standard TMB‐Horseradish peroxidase (HRP) kit (Figure S8, Supporting Information), and the amount of H_2_O_2_ produced was proportional to the concentration of AA (Figure S9, Supporting Information). In addition, the reaction products of AA catalyzed by OPA‐CsPbBr_3_ were determined by iodide method and ferrous oxidation in a xylenol orange (FOX) assay, respectively. OPA‐CsPbBr_3_ can catalyze AA to significantly change the color of the solution, further verifying the generation of H_2_O_2_ in the process (Figure S10, Supporting Information).

In order to further understand the reaction mechanism and performance of OPA‐CsPbBr_3_ as oxidase‐like enzymes for catalyzing oxidation of AA, DFT simulation was carried out. As shown in Figure 2F, combined with the free energy changes in the reaction process, the process and mechanism of CsPbBr_3_ catalyzing oxidation of AA were proposed. First, O_2_ adsorbs to the active site of the nanozyme to form the adsorbed intermediate. The adsorption energy was calculated as 8.48 kcal mol^−1^, which indicated that CsPbBr_3_ nanozyme had a strong adsorption capacity for O_2_. The strong adsorption capacity was conducive to a greater degree of elongation and fracture of O─O bond, which was attributed to the electron transfer through the electron‐pushing effect of the nanozyme during the adsorption process. The π* orbital of the anti‐bonding of O_2_ was filled and ROS of O_2_^•−^ was generated. And then, with the addition of the reactant AA, the AA would be further oxidized by O_2_^•−^ to produce H_2_O_2_. Based on the DFT calculation results, it confirmed that OPA‐CsPbBr_3_ NCs have high oxidase‐like activity, which can produce O_2_^•−^ by activating O_2_ and rapidly oxidizing AA to produce H_2_O_2_.

Furthermore, the ascorbate oxidase‐like catalytic activity of the OPA‐CsPbBr_3_ NCs was also explored. The steady‐state catalytic kinetics of AA oxidation was studied by recording the absorbance of AA as a function of time (Figures S11 and S12, Supporting Information), the calculated *K*_m_ (0.103 mm) value was lower and the *V*_max_ (2.303 × 10^−7^ m·s^−1^) was larger, which proved that OPA‐CsPbBr_3_ nanozyme had high catalytic activity for AA oxidation. In addition to catalytic activity, stability was another important index for the evaluation of nanozymes. As shown in Figure S13 (Supporting Information), after treating OPA‐CsPbBr_3_ nanozyme at different pH and temperature for 3 h, the relative change of absorption intensity at 259 nm was recorded after AA was catalyzed. It was found that the activity remained >80% after incubation at pH 2–10. At the temperature of 10–80 °C, the catalytic activity was not less than 93%. All the results showed that the OPA‐CsPbBr_3_ nanozyme displayed high stability.

### 2.3 Nanozyme Cascade Catalysis System Construction

**Figure 3:**
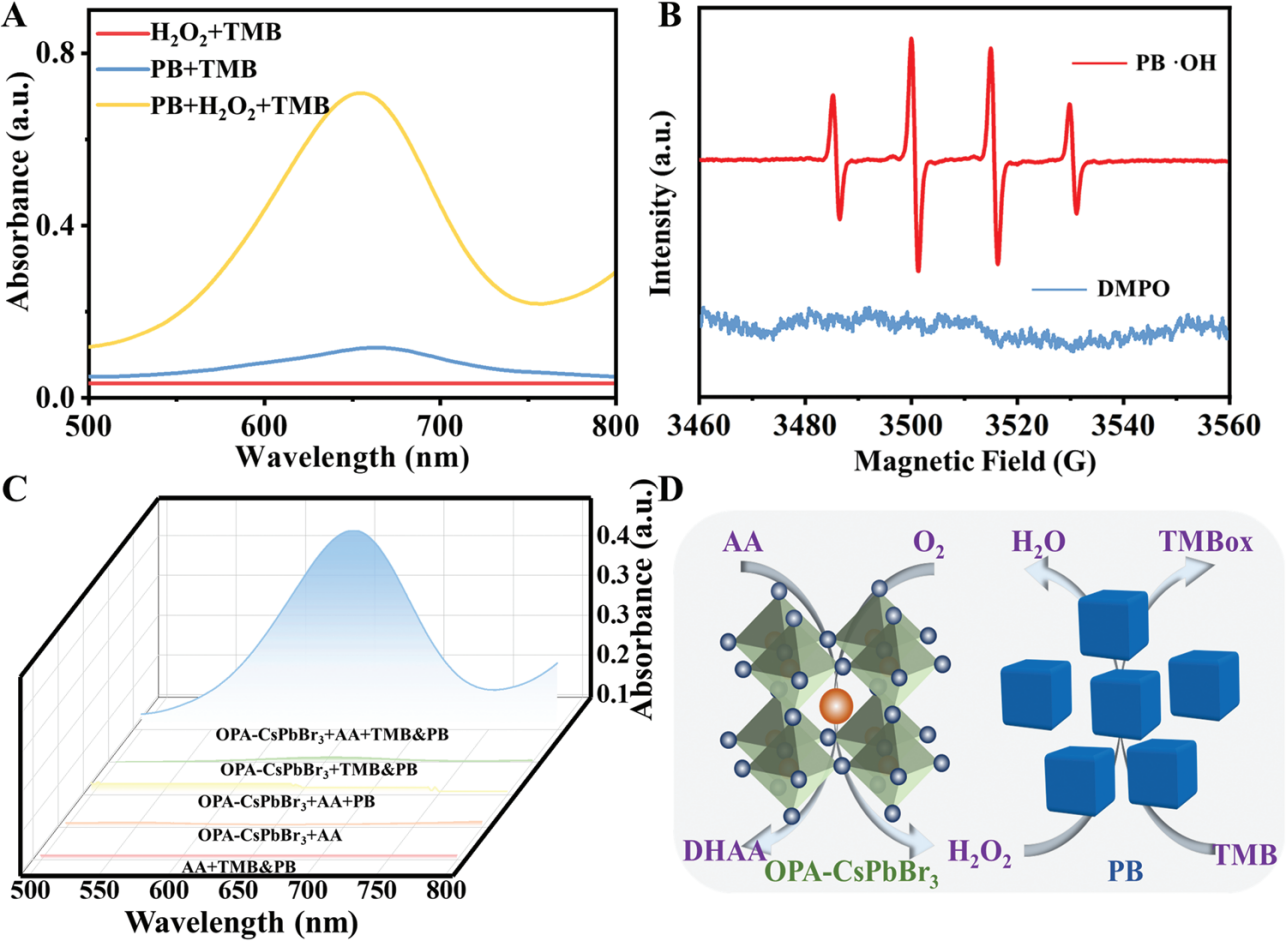
A) UV–vis absorption spectra of H_2_O_2_+TMB, PB+H_2_O_2_+TMB, and PB+ TMB system. B) EPR spectra of ^·^OH in PB catalytic reaction system. C) UV–vis absorption spectra of OPA‐CsPbBr_3_+AA+TMB&PB, OPA‐CsPbBr_3_+TMB&PB, OPA‐CsPbBr_3_+AA+PB, OPA‐CsPbBr_3_+AA, and AA+TMB&PB system. D) The reaction process of enzyme‐free cascade system constructed from OPA‐CsPbBr_3_ NCs and PB.

PB, as a common peroxidase‐like material, was selected to replace natural horseradish catalase to construct an enzyme‐free cascade system. It can be seen from the TEM image that the particle size of PB was small (≈10 nm) (Figure S14A, Supporting Information). The diffraction peaks at 24.7°, 35.4°, 39.7°, and 43.4° of the XRD pattern corresponded to 220, 400, 420, and 422 plane of the PB (Figure S14B, Supporting Information), which is consistent with previous reports.^[^ ^8^, ^17^ ^]^ UV absorption result further demonstrated the successful preparation of PB material (Figure S14C, Supporting Information). The appearance of an absorption peak ≈700 nm was attributed to the typical intermetallic charge transfer from Fe^2+^ to Fe^3+^ in the frame, which was the source of its peroxidase‐like activity. **Figure** **3**A shows the PB possessed peroxidase‐like activity. In addition, the UV–vis absorption spectra of TMB oxidized by different concentrations of H_2_O_2_ catalyzed by PB were recorded (Figure S15A, Supporting Information). It can be seen from Figure S15B (Supporting Information) that the peak intensity of TMBox increased linearly with the concentration of H_2_O_2_. The ROS generated by the H_2_O_2_ catalyzed by PB was tested as ^·^OH by EPR analysis (Figure 3B). The steady‐state kinetic and catalytic activity of PB was also studied by recording the UV absorption of PB‐TMB‐H_2_O_2_ with varying concentration of TMB (Figures S16 and S17, Supporting Information), the *K*_m_ and *V*_max_ value of PB nanoparticles was calculated to be 0.68 mm and 2.23 × 10^−6^ m·s^−1^, respectively. It indicated that the PB had high affinity and catalytic activity for TMB. All the above results demonstrated that PB, as a good nanozyme material, can be used to catalyze the generated H_2_O_2_ during the oxidation of AA for further cascade reaction. Based on the oxidase‐like activity of OPA‐CsPbBr_3_ nanozyme and peroxidase‐like activity of PB nanoparticles, an all‐nanozyme cascade system with high selectivity for AA was constructed. Subsequently, the feasibility of this cascade reaction was verified. After the OPA‐CsPbBr_3_ and AA were incubated at room temperature for 10 min, the supernatant was collected by centrifugation, and then PB and TMB were added to test the UV–vis spectrum of the solution. All the other control groups were carried out under this experimental condition. It can be seen from Figure 3C that only OPA‐CsPbBr_3_, AA, and PB participated in the reaction at the same time, the TMB can be oxidized to produce TMBox. The whole reaction system process was shown in Figure 3D. O_2_^•−^ produced by oxygen under the catalysis of OPA‐CsPbBr_3_ can catalyze AA to produce H_2_O_2_, and the product H_2_O_2_ was further catalyzed by PB to produce ^·^OH, which oxidizes TMB to TMBox. (Equations (5), (6), (7)).

(5) $$O_{2}^{\cdot -}+\mathrm{AA}=\mathrm{DHAA}+H_{2}O_{2}$$

(6) $$H_{2}O_{2}+\mathrm{PB}\to \cdot \mathrm{OH}$$

(7) ·OH+TMB→TMBox

### 2.4 Nanozyme Cascade Reaction for Dual‐Mode Analysis

**Figure 4:**
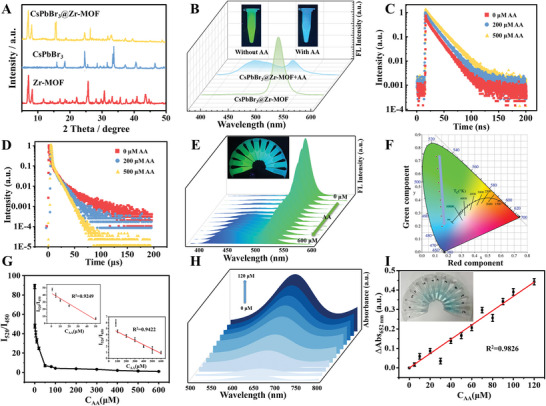
A) XRD spectra of CsPbBr_3_, Zr‐MOF, and CsPbBr_3_@Zr‐MOF nanocomposite. B) Fluorescence spectra of CsPbBr_3_@Zr‐MOF nanocomposite before and after the addition of AA (inset was the actual fluorescent images under 365 nm UV light irradiation). Time‐resolved FL decay curves of C) Zr‐MOF at 450 nm and D) CsPbBr_3_ at 520 nm for CsPbBr_3_@Zr‐MOF nanocomposite before and after adding different concentration of AA. E) Fluorescence emission spectra of CsPbBr_3_@Zr‐MOF nanocomposite after addition of different concentrations of AA and the fluorescent images of the solution. F) Corresponding CIE chromaticity diagram. G) The fitting curve between *I*_520_/*I*_450_ and the AA concentration. H) UV–vis spectra of TMBox at different AA concentrations using the nanozyme cascade system. I) The fitting curve between the absorption peak intensity of TMBox at 652 nm and AA concentration.

Above results indicated that the catalytic activity of OPA‐CsPbBr_3_ NCs can be utilized to construct a cascade reaction for the establishment of a colorimetric sensing system for AA. Besides possessing high catalytic activity, OPA‐CsPbBr_3_ NCs also displayed high fluorescent characteristics. The stability of OPA‐CsPbBr_3_ NCs in HAc‐NaAc buffer was studied by recording the fluorescence spectra after storage for different periods (Figure S3, Supporting Information). The results show that the fluorescence intensity of OPA‐CsPbBr_3_ NCs can still maintain ≈80% of the initial intensity after being stored in HAc‐NaAc buffer for 10 days, suggesting the good stability of the NCs. Furthermore, a ratiometric fluorescence sensing system for AA by combing fluorescent OPA‐CsPbBr_3_ with Zr‐MOF was constructed. First, the as‐synthesized OPA‐CsPbBr_3_ was combined with Zr‐BDC‐NH_2_ by the adsorption interaction to prepare the nanocomposite with high fluorescence characteristics. The morphologies and structures of the synthesized CsPbBr_3_@Zr‐MOF were characterized by scanning electron microscope (SEM), XRD, and FT‐IR analysis. As shown in Figure S18A (Supporting Information), the Zr‐MOF crystals showed an octahedral geometry. When OPA‐CsPbBr_3_ NCs were composited with Zr‐MOF, CsPbBr_3_ crystal material could be observed loading on the surface of Zr‐MOF, and the original morphology of Zr‐MOF did not change significantly (Figure S18B, Supporting Information). From the analysis results of XRD (**Figure** **4**A), it can be seen that the diffraction peaks of the XRD pattern of Zr‐MOF are sharp, which proves the high crystallinity of the material. When CsPbBr_3_ was incorporated, the diffraction peaks of the nanocomposites can be well matched with that of Zr‐MOF and CsPbBr_3_. In addition, the FT‐IR spectra of CsPbBr_3_@Zr‐MOF showed the main absorption peaks of CsPbBr_3_ and Zr‐MOF (Figure S18C, Supporting Information). All above results confirmed the successful preparation of the nanocomposites. Furthermore, the fluorescence properties of the CsPbBr_3_@Zr‐MOF nanocomposite were studied, the fluorescence spectra of the composites doped with different amounts of MOF were recorded. As the amount of MOF gradually increased, the fluorescence intensity of CsPbBr_3_ gradually increased (Figure S18D, Supporting Information). To investigate the mechanism for the increase of the fluorescence intensity for CsPbBr_3_ after the introduction of Zr‐MOF, the fluorescence lifetime analysis was performed. The fluorescence lifetime values of CsPbBr_3_, CsPbBr_3_@Zr‐MOF (250 µg mL^−1^), and CsPbBr_3_@Zr‐MOF (500 µg mL^−1^) were calculated as 10.57, 13.19, and 15.78 µs, respectively. It indicated that the fluorescence lifetime of CsPbBr_3_ gradually enhanced when the amount of MOF increased (Figure S18E, Supporting Information). Furthermore, the ratio of the faster component *τ*_1_ of CsPbBr_3_, CsPbBr_3_@Zr‐MOF (250 µg mL^−1^), and CsPbBr_3_@Zr‐MOF (500 µg mL^−1^) was calculated to be 71.53%, 79.66%, and 82.23%, respectively. It demonstrated the short‐lived lifetime (*τ*_1_) also increased after incorporation of MOF. The increase in the ratio of *τ*_1_ indicated that the radiative transition of excitons from conduction band to valence band plays a dominant role.^[^ ^18^ ^]^ This was attributed to the passivation effect of Zr‐MOF on the surface defects of OPA‐CsPbBr_3_ NCs, which reduced the non‐radiative transition of CsPbBr_3_ and enhanced its fluorescence properties.^[^ ^19^ ^]^ It indicated the protective effect of MOF material inhibited the original electronic transition and cavitation of CsPbBr_3_, which reduced the fluorescence quenching effect caused by the aggregation of the material and effectively improved the fluorescence performance of CsPbBr_3_.^[^ ^20^ ^]^

Subsequently, the fluorescence change of the CsPbBr_3_@Zr‐MOF solution after addition of AA was recorded. The blue fluorescence emission peak attributed to the ligand of Zr‐MOF at 450 nm greatly enhanced, while the green fluorescence peak of CsPbBr_3_ at 520 nm significantly weakened, at this time, the fluorescence color of the solution changed from green to blue under UV light irradiation (Figure 4B). Therefore, a ratiometric fluorescent sensor based on the CsPbBr_3_@Zr‐MOF nanocomposite was constructed for detection of AA. In order to further study the sensing mechanism, the fluorescence lifetime of the two fluorescence peaks of the nanocomposite before and after adding different concentrations of AA was monitored, it was found that with the increase of AA concentration, the fluorescence lifetime of Zr‐MOF obtained by double exponential function fitting increased from 8.08 to 17.37 ns (Figure 4C), while the fluorescence lifetime of CsPbBr_3_ decreased from 13.19 to 10.12 µs (Figure 4D). This is mainly because the large specific surface area of Zr‐MOF is favorable for AA binding, and the strong interaction between AA and Zr─O clusters will destroy the crystal structure of Zr‐MOF and release the ligand BDC‐NH_2_, thus enhancing the fluorescence intensity of the ligand.^[^ ^21^ ^]^ TEM analysis further demonstrated the structure destruction of Zr‐MOF (Figure S19A, Supporting Information). At the same time, CsPbBr_3_ lost the protective effect of Zr‐MOF, and its fluorescence intensity and fluorescence lifetime decreased to that before doping. Meanwhile, the nanocomposite synthesized under different amounts of Zr‐MOF was used to test its analytical performance for AA detection. By comparing the fluorescence intensity ratios (*I*_520_/*I*_450_) of the solutions before and after the addition of AA (Figure S19B, Supporting Information), it was found that the fluorescence intensity ratio variation showed the largest when the amount of Zr‐MOF was 250 µg mL^−1^. Therefore, nanocomposite synthesized at this ratio was selected for subsequent sensing applications.

Based on the feasibility of CsPbBr_3_@Zr‐MOF nanocomposite for ratiometric fluorescent sensing of AA, the analytical performance of the sensor was further evaluated. As can be seen from Figure 4E, with the concentration of AA increasing, the fluorescence intensity of Zr‐MOF at 450 nm gradually increased, while the fluorescence intensity of CsPbBr_3_ at 520 nm gradually weakened. The corresponding CIE chromaticity diagram showed obvious color changes with the concentration of AA increasing (Figure 4F). The fluorescence intensity ratio *I*_520_/*I*_450_ showed a good linear relationship with the AA concentration in the range of 1–50 and 100–600 µm with the correlation coefficients *R* ^2^ of 0.925 and 0.942, respectively (Figure 4G), and the limit of detection (LOD) was estimated to be 0.36 µm. Based on the ascorbate oxidase‐like activity of OPA‐CsPbBr_3_, we continued to explore the feasibility of CsPbBr_3_@Zr‐MOF for colorimetric quantitative detection of AA. By comparing the catalytic performance of CsPbBr_3_ and CsPbBr_3_@Zr‐MOF, it was found that the activity of catalyzing AA to generate H_2_O_2_ was not affected after combing CsPbBr_3_ with MOF material (Figure S20, Supporting Information). Therefore, using two nanozyme materials, CsPbBr_3_@Zr‐MOF and PB, a nanozyme cascade system was constructed for quantitative colorimetric detection of AA. It can be seen from Figure 4H that the UV absorption peak of TMBox gradually increased with the concentration of AA increasing. A good linear relationship was presented in the range of 0–120 µm (Figure 4I) with the *R* ^2^ of 0.983, and the LOD was calculated to be 1.61 µm.

The selectivity of the ratiometric fluorescent and colorimetric dual‐mode sensor for detection of AA was investigated by monitoring the fluorescence intensity ratio of CsPbBr_3_@Zr‐MOF and the UV absorption peak of the product TMBox after adding various interfering biomolecules and AA. As shown in Figure S21A,B (Supporting Information), except for AA, the addition of other biomolecules (including dopamine, glutathione, cysteine, glucose, cholesterol, lysine, arginine, serine, proline, alanine, glycine, phenylalanine, leucine, histidine, urea, melamine, tyrosine, and isoleucine) did not produce obvious fluorescence changes. In addition, for the colorimetric detection, except that AA can be catalyzed to generate H_2_O_2_, which further generates the absorption signal of TMBox, the other eighteen biomolecules could not generate signals (Figure S21C,D, Supporting Information). Therefore, the high selectivity of the dual‐mode sensor for detection of AA was demonstrated. In order to evaluate the application potential of this ratiometric fluorescent and colorimetric dual‐mode sensor in practical detection, the standard addition method was used to monitor the concentration of AA in human serum samples. First, the serum was diluted for 10‐fold and CsPbBr_3_@Zr‐MOF nanocomposite was added to the solution. And then a certain amount of AA (20, 50, and 100 µm) was added to the serum sample. Subsequently, the fluorescence and UV–vis absorption spectra were recorded, respectively (Figure S22, Supporting Information). It can be seen from Table S3 (Supporting Information) that the original content of AA in human serum was measured ≈24.2 and 25.1 µm by this dual‐mode sensor, which was consistent with the value reported in the literature.^[^ ^22^ ^]^ The recoveries for using fluorescence and colorimetric method were between 96.7–108.4% and 99.3–106.4%, respectively. These results demonstrate the good accuracy and practicality of the dual‐mode sensor for fluorescence and colorimetric sensing of AA in human serum samples.

### 2.5 POC Analysis

**Figure 5:**
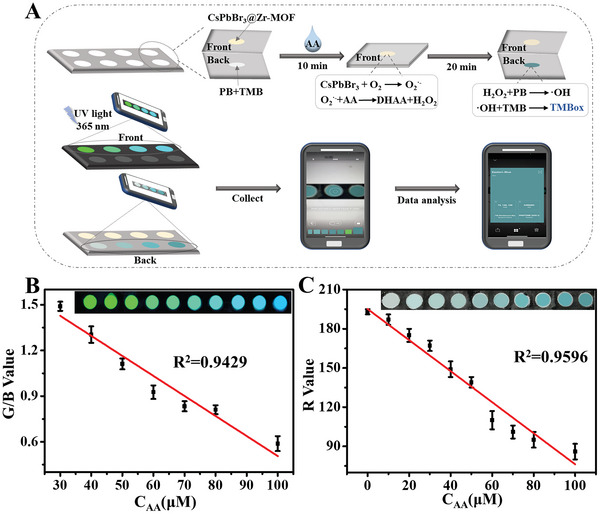
A) Schematic diagram of the preparation and testing process of a portable paper‐based device. B) The relationship between the fluorescent intensity ratio (G/B) value and AA concentration. Inset: the actual fluorescent image of the paper‐based circular well device under 365 nm UV light irradiation. C) The relationship between the colorimetric intensity (R) value and AA concentration. Inset: the image of the paper‐based circular well device.

To investigate the potential of this dual‐mode sensor for visual point‐of‐care detection, a portable, low‐cost test strip of the paper‐based device for the detection of AA was prepared. **Figure** **5**A shows the preparation process for the device. First, a uniform circular hole paper‐based device was prepared by a simple waxy screen‐printing technique, and the synthesized CsPbBr_3_@Zr‐MOF and PB‐TMB were coated on the upper and lower holes of the paper‐based device, respectively. Different concentrations of AA solution were dropped into the paper‐based circular wells, in which the front side was coated with CsPbBr_3_@Zr‐MOF nanocomposites. After 10 min of reaction, the paper base was folded in half. At this time, the reaction product H_2_O_2_ penetrated into the lower paper base with the buffer, and further reacted with the PB‐TMB system in the lower layer, thereby producing a blue product of TMBox. The fluorescent color of the upper layer of paper base could be clearly observed to change from green to blue under 365 nm UV lamp irradiation, while the lower layer of paper base showed a color change from colorless to dark blue. Then, shoot with a smartphone, and the captured image was processed in RGB color space using a smartphone app (Swatches). As shown in Figure 5B, the fluorescence color signal was converted to G/B value ratio, the G/B value showed a good linear correlation with the AA concentration (30–100 µm) (G/B = −0.0137C_AA_+1.823, *R* ^2^ = 0.943) and the LOD was calculated as low as 10.68 µm. In addition, choosing the ordinate and abscissa as the red (R) value and AA concentration, the R color intensity of the colorimetric color signal has a linear relationship with the AA concentration (0–100 µm) (Figure 5C). The fitted equation was *R* = −1.226C_AA_+196.6 with the *R* ^2^ of 0.960, and the LOD was calculated as low as 4.01 µm. These results demonstrated the successful fabrication of a smartphone‐based dual‐mode biosensor with the advantages of portability, visualization, and low‐cost that can be used for high‐throughput and POC disease analysis.

## 3 Conclusion

In summary, OPA‐CsPbBr_3_ NCs with oxidase‐like activity were applied in biomimetic cascade catalysis for dual‐mode biosensing. First, OPA‐CsPbBr_3_ NCs with good water stability were successfully prepared through an amphiphilic polymer ligand‐assisted reprecipitation method. In addition, the oxidase‐like and ascorbate oxidase‐like of OPA‐CsPbBr_3_ NCs were discovered. The mechanism research verified that O_2_ was activated by the OPA‐CsPbBr_3_ nanozyme to generate O_2_^•−^ via the oxidase‐like pathway, then the O_2_^•−^ directly oxidizes AA and produces H_2_O_2_. Next, multifunctional CsPbBr_3_@Zr‐MOF with oxidase‐like activity was cascaded with PB with peroxidase‐like activity to construct a nanozyme cascade system for realizing the dual mode (colorimetric and ratiometric fluorescent) biosensing. Combined with a smartphone‐based paper POC device, the facile, rapid, sensitive, and accurate detection of neurochemical marker was demonstrated. The high stability and nanozyme activity of CsPbX_3_ perovskite NCs make them can be regarded as good candidates for biocatalytic analysis, which opens a new avenue for in vitro disease diagnostic. Our future work will mainly focus on two aspects: i) the selectivity and activity of the perovskite nanozymes remain to be enhanced when they are working in presence of multiple substrates. To further improve the nanozyme activities, the strategies of hybriding with other metal nanoparticles, doping with metal atoms could be proposed. In addition, tuning exposed lattice plane by changing the crystals structure could be a better choice for improving the selectivity of the perovskite NCs. ii) The OPA‐CsPbBr_3_ NCs with oxidase‐like and ascorbate oxidase‐like activities will be applied in the field of AA oxidation induced cancer therapy and other biomedical fields.

## 4 Experimental Section

### Materials and Reagents

TMB was bought from Sigma–Aldrich. HRP was bought from BBI Life Sciences. *N, N*‐dimethylformamide (DMF), toluene, methanol, *n*‐hexane, H_2_O_2_ (30%), and AA were purchased from Sinopharm Chemical Reagent Co., Ltd. (Shanghai, China). Cesium bromide (CsBr), lead bromide (PbBr_2_), and 2‐amino‐benzenedicarboxylic acid (NH_2_‐BDC) were purchased from Aladdin. Zirconium tetrachloride (ZrCl_4_) and OAm were purchased from Shanghai Macklin Biochemical Co., Ltd. The water used in all experiments was ultrapure water. All chemicals and reagents were analytical grade and used without further purification.

### Apparatus

TEM images were captured on the JEM‐2100 transmission electron microscope. The HRTEM images were captured on a Tecnai G2 F30 transmission electron microscope (acceleration voltage of 300 kV). The scanning electron microscope (Zeiss_Supra55, Carl Zeiss AG, Germany) was used to capture SEM images. UV–vis absorption spectrum was performed on a UV2550 spectrometer (SHIMADZU). The fluorescence emission spectrum was scanned by UV–vis/near‐infrared fluorescence spectrometer (FS5, Edinburgh Instruments Ltd.). X‐ray diffractometer D8ADVANCE (Bruker Co.) was used for XRD analysis. FT‐IR spectroscopy was surveyed by an Antaris II Fourier transform near‐infrared spectrometer. The material was analyzed by XPS using a Thermo Scientific ESCALAB 250Xi X‐ray photoelectron spectrometer. FLS 1000 Fluorometer (Edinburgh Instruments Ltd.) was used to measure the decay of the fluorescence lifetime. Determination of specific surface area of nanomaterials was performed by BET method (Rubothem, Germany). EPR signals were recorded by an A300‐10/12 EPR spectrometer (Bruker, Germany). ESI‐MS was performed with Bruker Dalton maXis instrument (Bruker, Germany).

### Preparation of OPA‐CsPbBr_3_ NCs

The OPA‐CsPbBr_3_ NCs were prepared according to our previous report using a room‐temperature synthesis method.^[^ ^13^ ^]^ First, CsBr (0.4 mmol) and PbBr_2_ (0.4 mmol) were added to DMF (10 mL) and stirred until the solid was completely dissolved to obtain the perovskite precursor. OAm (50 µL) and amphiphilic OPA (2 mg) polymers synthesized according to the previous report were added to the precursor solution (1 mL). After stirring at room temperature for 5 min, the above mixture was poured into toluene (20 mL) and stirred for 10 min, then centrifuged at 9000 rpm for 5 min, and washed twice with toluene. The precipitate was dried under vacuum for 2 h to obtain OPA‐CsPbBr_3_ NCs.

### Preparation of Zr‐MOF

The Zr‐MOF composite was prepared as follows. BDC‐NH_2_ (1.83 mmol) and ZrCl_4_ (1.56 mmol) were added in DMF (30 mL) and then transferred to a 50 mL Teflon‐lined stainless steel autoclave and reacting at 120 °C for 24 h. After the reaction, the products were separated by centrifugation and washed with DMF/methanol for several times. Then it was dried in a vacuum oven at 80 °C for 12 h.^[^ ^23^ ^]^

### Preparation of CsPbBr_3_@Zr‐MOF

OPA‐CsPbBr_3_ (15 mg) was added to *n*‐hexane (10 mL) and stirred magnetically to form a homogeneous solution. Then, 0.25, 0.5, 1, 2.5, and 5 mg of Zr‐MOF were added, respectively, and after magnetic stirring for 2 h, the product was centrifugated at 9000 rpm for 5 min, followed by vacuum drying at 60 °C for 2 h to obtain CsPbBr_3_@Zr‐MOF nanocomposite.

### Oxidase‐Like Activity of OPA‐CsPbBr_3_ NCs

OPA‐CsPbBr_3_ (1 mg) was dissolved in HAc‐NaAc buffer (980 µL, pH 4), and TMB (20 µL, 50 mm) was added. After reacting at room temperature for 20 min, the absorption peak of TMB oxidation product (TMBox) at 652 nm was measured by UV–vis spectrophotometer.

### Catalytic Oxidation of AA by OPA‐CsPbBr_3_ NCs

The method for the catalytic oxidation of AA by OPA‐CsPbBr_3_ NCs was performed by adding OPA‐CsPbBr_3_ (1 mg) in HAc‐NaAc buffer solution (900 µL, pH 4) containing different concentrations of AA (0, 5, 10, 15, 20, 30, 40, 50, and 60 µm), the mixture was incubated at room temperature for 10 min and then centrifuged at 9000 rpm for 5 min to remove CsPbBr_3_. Afterward, TMB (20 µL, 50 mm) and HRP (20 µL, 0.1 mg mL^−1^) were added into the supernatant. After incubated for 15 min, the absorption peak of TMBox at 652 nm was measured by UV–vis spectrophotometer.

To evaluate the catalytic stability of OPA‐CsPbBr_3_ nanozyme under harsh conditions, OPA‐CsPbBr_3_ NCs solution was incubated under different pH and temperature for 3 h. After that, OPA‐CsPbBr_3_ NCs (100 µL, 10 mg mL^−1^) were added into AA solution (900 µL, 50 µm) and incubated at room temperature for 10 min. The relative change of AA absorption intensity at 259 nm was recorded to indicate the relative catalytic activity of OPA‐CsPbBr_3_ nanozyme.

### Detection of H_2_O_2_ by Iodide Method and FOX Assay

First, OPA‐CsPbBr_3_ (1 mg) was added to HAc‐NaAc buffer (900 µL, pH = 4) containing two different concentrations of AA (100 µL, 50/100 µm) and incubated at room temperature for 10 min, and then centrifuged at 9000 rpm for 5 min to remove OPA‐CsPbBr_3_. Then the supernatant (500 µL) was added to potassium iodide (KI) solution (400 µL, 1 mol L^−1^) and ammonium molybdate solution (100 µL, 0.01 mol L^−1^). After 30 min, the absorption of the above solution was tested using UV–vis spectroscopy.

The pretreatment conditions of FOX assay were the same as those of iodide method above, except that the supernatant (500 µL) was added with 500 µL FOX reagent, which contains (NH_4_)_2_Fe(SO_4_)_2_ (250 µm), xylenol orange (100 µm), and H_2_SO_4_ solution (25 mm). After 30 min, the absorption of the above solution was tested using UV–vis spectroscopy.

### Catalytic Oxidation of TMB by PB Nanozyme

The catalytic oxidation of TMB by PB nanozyme was carried out by adding PB (10 µL, 1 mg mL^−1^) into HAc‐NaAc buffer (970 µL, pH 4) containing different concentrations of H_2_O_2_ (0–25 µm) and TMB (20 µL, 50 mm). Then, the mixture was incubated at 35 °C for 30 min, and the absorption intensity of TMBox was measured.

### Construction of Nanozymes Cascade Reaction System

A typical cascade reaction was prepared using OPA‐CsPbBr_3_ and PB as nanozymes. First, OPA‐CsPbBr_3_ (1 mg) was added into HAc‐NaAc buffer (1 mL, pH 4) containing AA (100 µm), incubated at room temperature for 10 min, and then centrifuged at 9000 rpm for 5 min to remove CsPbBr_3_. TMB (20 µL, 50 mm) and PB (10 µL, 1 mg mL^−1^) were added dropwise to the supernatant. Then, the above solution was incubated at 35 °C for 30 min, and the reaction solution was measured by UV–vis spectrophotometer.

### Colorimetric Detection of AA by CsPbBr_3_@Zr‐MOF

CsPbBr_3_@Zr‐MOF (1.5 mg) was added in HAc‐NaAc buffer (1 mL, pH 4) containing different concentrations of AA (0–120 µm), incubated at room temperature for 10 min, and then centrifuged at 9000 rpm for 5 min to remove CsPbBr_3_@Zr‐MOF. TMB (20 µL, 50 mm) and PB (10 µL, 1 mg mL^−1^) were added dropwise to the supernatant. Then, the above solution was incubated at 35 °C for 30 min, and the reaction solution was measured by UV–vis spectrophotometer. In order to determine the selectivity of CsPbBr_3_@Zr‐MOF for AA colorimetric detection, AA and other biomolecules with concentration of 100 µM were added for testing. The procedures were the same as above. All tests were repeated three times at room temperature.

### Ratiometric Fluorescence Detection of AA by CsPbBr_3_@Zr‐MOF

CsPbBr_3_@Zr‐MOF (1.5 mg) was added in HAc‐NaAc buffer (1 mL, pH 4) containing different concentrations of AA (0–600 µm). After incubation for 5 min, the luminescence peaks at 450 and 520 nm of CsPbBr_3_@Zr‐MOF with different concentrations of AA were recorded under excitation at 328 nm, and the color of the samples was observed under UV light. To determine the selectivity of CsPbBr_3_@Zr‐MOF for AA fluorescence detection, AA and other biomolecules (100 µM) were added for detection. The procedures were the same as above.

### Determination of AA in Human Serum Sample

Clinical serum sample from healthy donor was supplied by Subei People's Hospital of Jiangsu province. The serum sample was approved by the ethics committees of Yangzhou University (YXYLL‐2023‐104). The serum sample was analyzed after a final 10‐fold dilution. The experimental conditions and assay procedures were the same as the AA standard analytical assay described above, except that the standard target solution was replaced with the real sample. Colorimetric and fluorescent signals were monitored for replicate samples (*n* = 3). In addition, a certain concentration of standard AA solution was added to the above diluted human serum sample to verify the accuracy of the method.

### Cascade Reactions of Nanozymes in Paper‐Based Devices

Paper‐based devices were fabricated using a simple wax screen printing technique. The two nanozymes CsPbBr_3_@Zr‐MOF and PB were immobilized on the upper and lower layer of the paper‐based device, respectively. First, HAc‐NaAc buffer containing different concentrations of AA was dropped onto a paper‐based circular hole coated with CsPbBr_3_@Zr‐MOF material, and the fluorescence image was recorded under a 365 nm UV lamp with a smartphone. Then the paper base was folded in half after incubating at room temperature for 10 min, and the supernatant was soaked into the circular hole of the paper base coated with PB in the lower layer. Then TMB was dropped, and the physical image of the TMBox product was recorded with a smartphone. Finally, the Swatches App in the smartphone was used to perform RGB (red, green, and blue) color numerical processing for the captured physical image. According to the standard curve of RGB value with AA concentration, the real‐time monitoring of AA by smartphone was realized.

## Conflict of Interest

The authors declare no conflict of interest.

## Supporting information

Supporting Information

## Data Availability

The data that support the findings of this study are available from the corresponding author upon reasonable request.
