# Supplementary material for: Amphiphilic Polymer Capped Perovskite Compositing with Nano Zr‐MOF for Nanozyme‐Involved Biomimetic Cascade Catalysis
Source: Adv Sci (Weinh). 2023 Aug 27;10(31):2304149. doi: 10.1002/advs.202304149 (PMC10625115; doi:10.1002/advs.202304149)
Supplement: Supplementary file 1 — Supporting Information [file ADVS-10-2304149-s001.pdf]

ADVANCED SCIENCE
Open Access

Supporting Information

for *Adv. Sci.*, DOI 10.1002/advs.202304149

Amphiphilic Polymer Capped Perovskite Compositing with Nano Zr-MOF for Nanozyme-Involved Biomimetic Cascade Catalysis

Qiuyu Ye, Enxian Yuan, Jin Shen, Mingli Ye, Qin Xu, Xiaoya Hu, Yun Shu and Huan Pang**

Supporting Information

Amphiphilic Polymer Capped Perovskite Compositing with Nano Zr-MOF for Nanozyme-Involved Biomimetic Cascade Catalysis

Qiuyu Ye, Enxian Yuan, Jin Shen, Mingli Ye, Qin Xu, Xiaoya Hu, Yun Shu, Huan Pang**

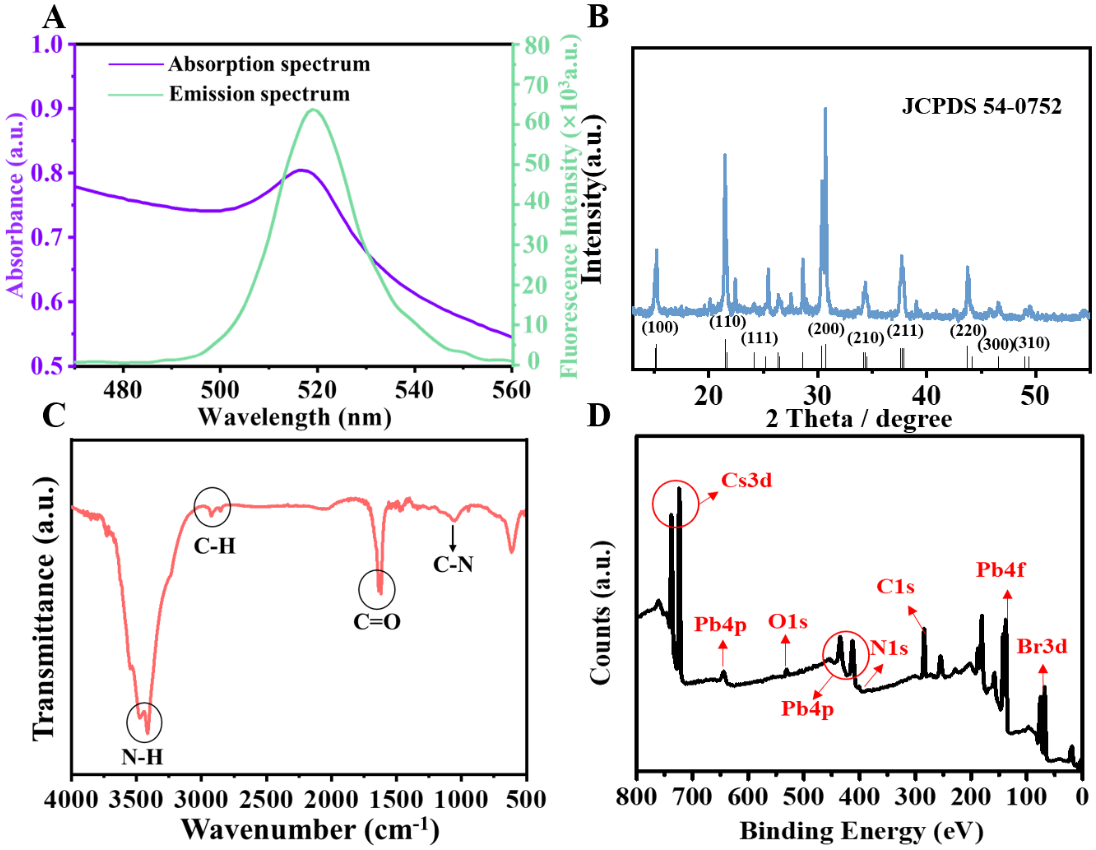

Figure S1. (A) UV-vis absorption (purple) and fluorescence (green) spectra of OPA-$CsPbBr_3$ NCs. (B) XRD, (C) FT-IR, and (D) XPS spectra of OPA-$CsPbBr_3$ NCs.

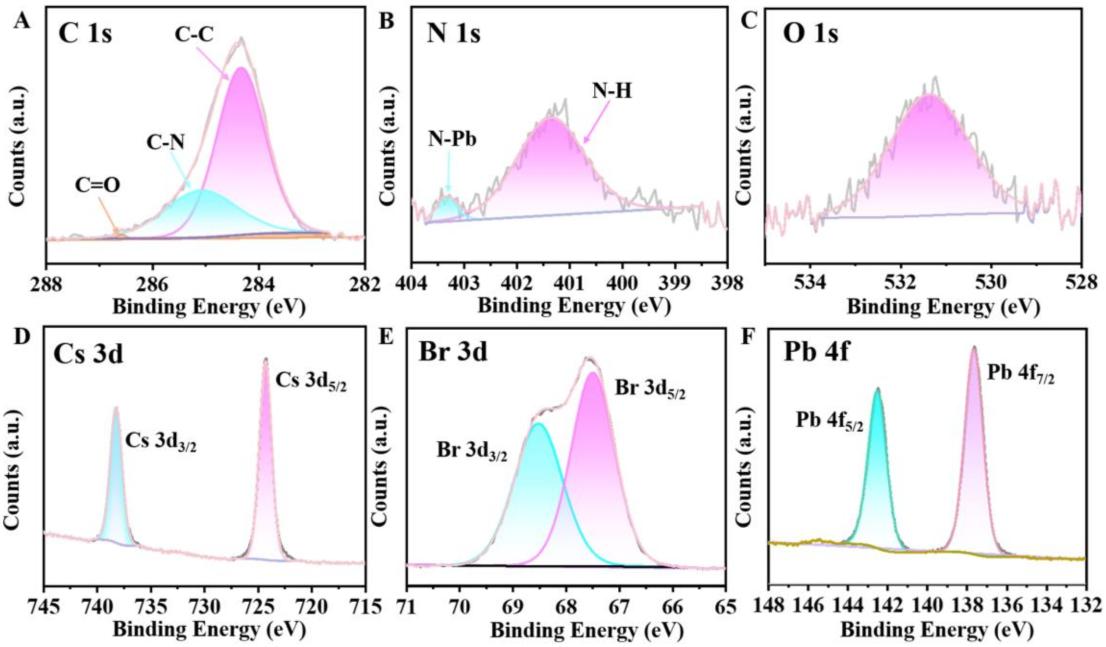

Figure S2. (A) C 1s, (B) N 1s, (C) O 1s, (D) Cs 3d, (E) Br 3d, (F) Pb 4f XPS spectra of OPA-$CsPbBr_3$ NCs.

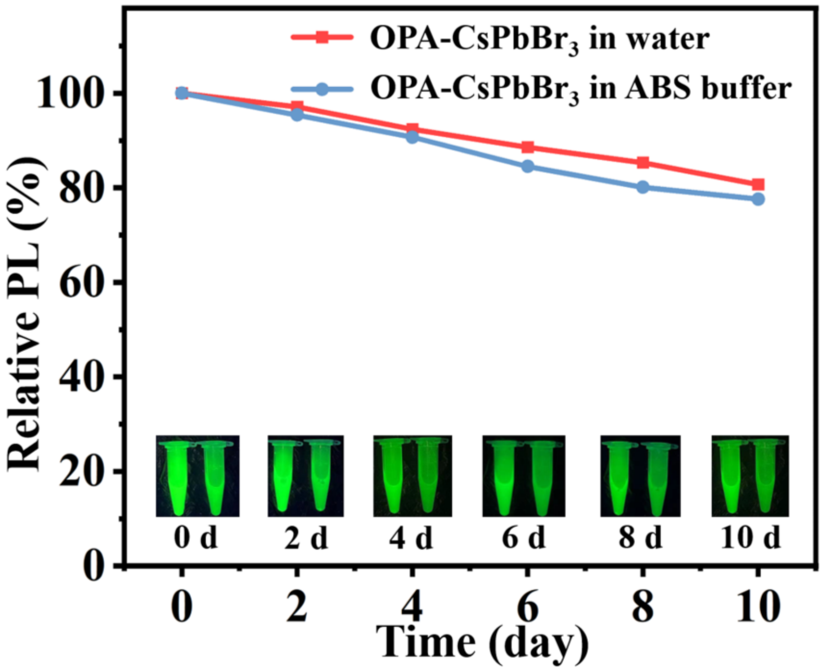

Figure S3. Fluorescent stability diagrams of OPA-$CsPbBr_3$ NCs in water and HAc-NaAc buffer (pH=4). The inset shows the optical images of OPA-$CsPbBr_3$ NCs in water (left) and HAc-NaAc buffer (right) under 365 nm UV light at different time periods.

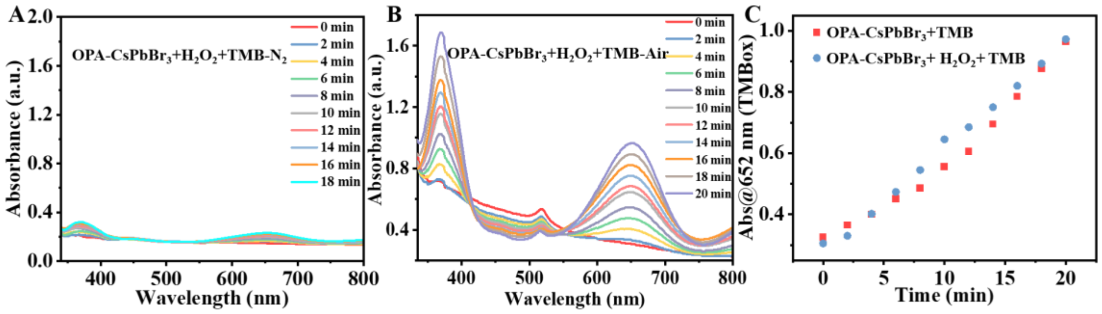

Figure S4. (A) Real-time UV-vis absorption spectra of the OPA-$CsPbBr_3$+H_2O_2+TMB system under N_2 environment. (B) Real-time UV-vis absorption spectra of the OPA-$CsPbBr_3$+H_2O_2+TMB system under air environment. (C) Time-dependent UV-vis absorbance of TMBox at 652 nm by OPA-$CsPbBr_3$ NCs catalyzing TMB in absence and presence of H_2O_2, respectively.

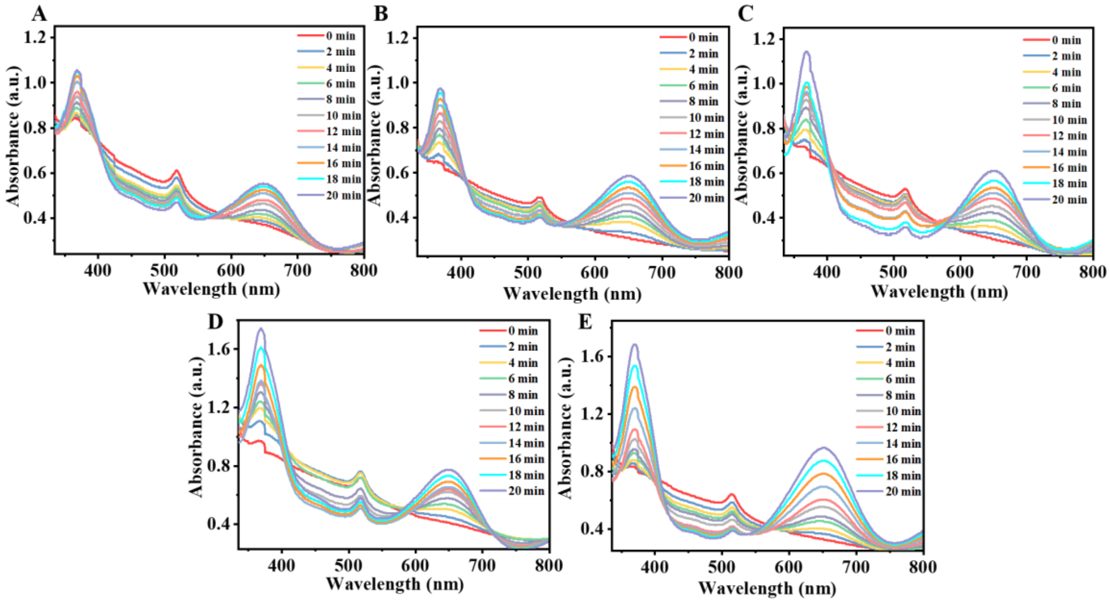

Figure S5. Real-time UV-vis absorption spectra of the catalyzed oxidation of different concentrations of TMB through the oxidase-like activity by OPA-$CsPbBr_3$ NCs. (A) 0.2 mM, (B) 0.4 mM, (C) 0.6 mM, (D) 0.8 mM, (E) 1.0 mM.

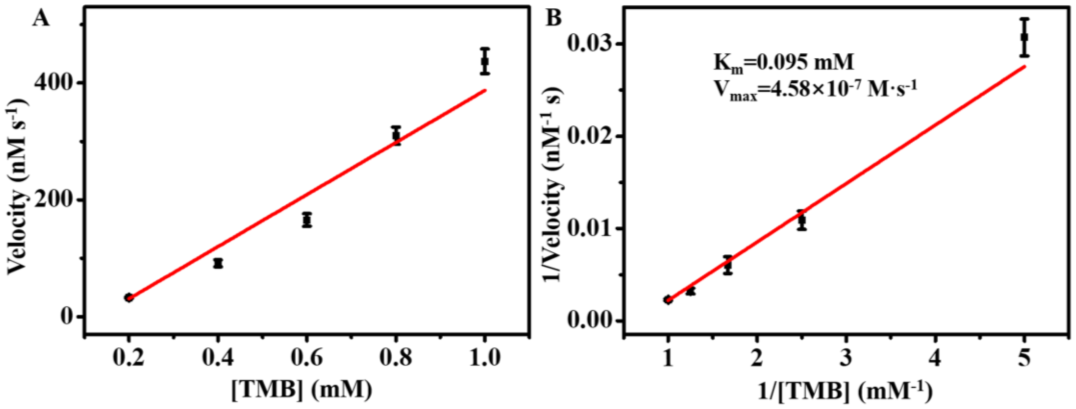

Figure S6. Steady-state kinetics for OPA-$CsPbBr_3$ catalytic oxidation of TMB in buffer (HAc-NaAc, pH = 4). (A) The Velocity as a function of [TMB]. (B) The Lineweaver-Burk plot.

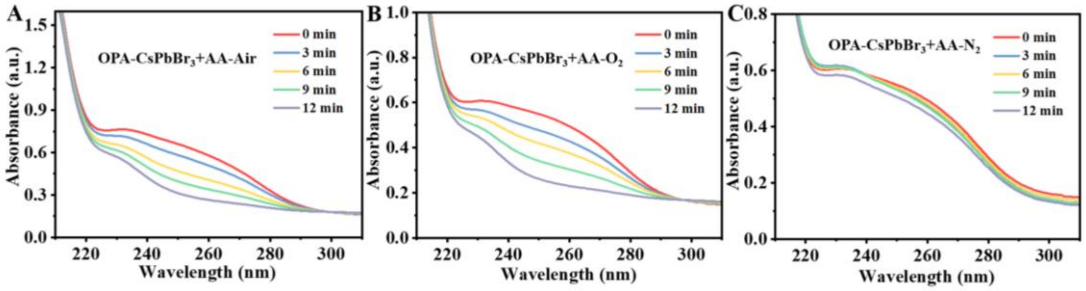

Figure S7. Real-time UV absorption spectra of AA after autooxidation catalyzed by OPA-$CsPbBr_3$ nanozyme under (A) Air, (B) O_2 and (C) N_2 environment, respectively.

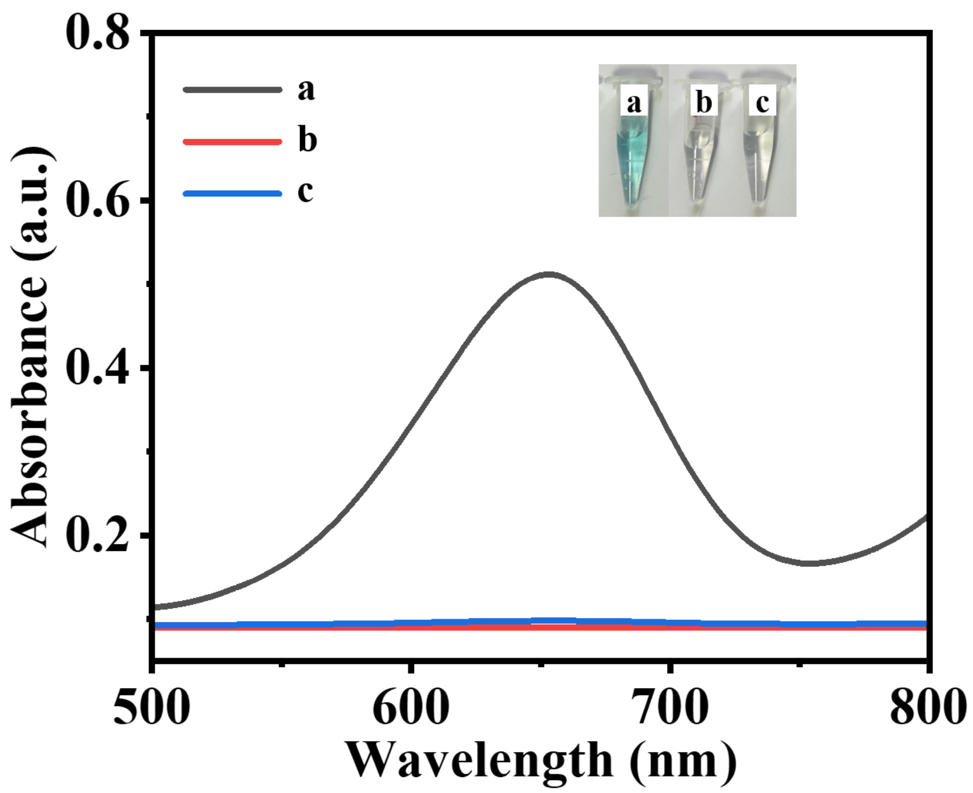

Figure S8. UV-vis absorption spectra of OPA-$CsPbBr_3$+AA+TMB&HRP (a), AA+TMB&HRP (b) and OPA-$CsPbBr_3$+AA+TMB (c).

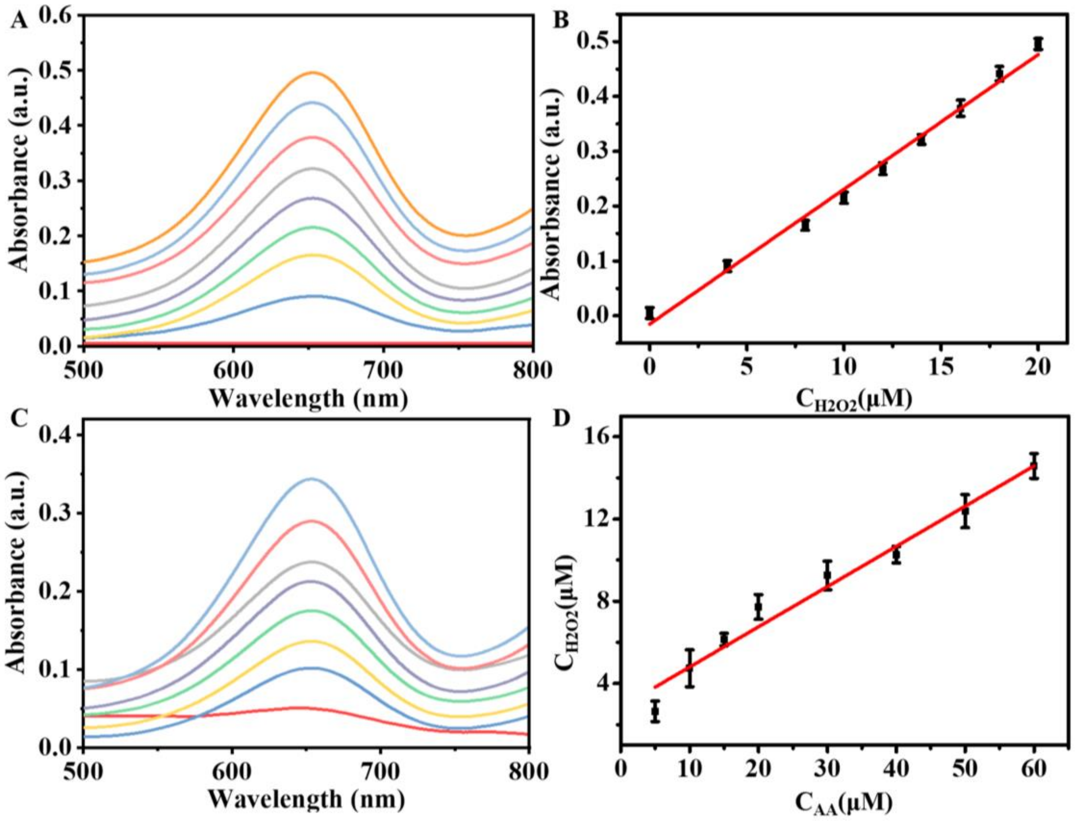

Figure S9. (A) UV-vis absorption spectra of different concentrations of H_2O_2 added to TMB-HRP solution. (B) Standard calibration plot for detection of H_2O_2 by the colorimetric TMB-HRP method. (C) UV-vis absorption spectra of H_2O_2-TMB-HRP solution, in which the H_2O_2 was produced by OPA-$CsPbBr_3$ catalytic oxidation of different concentrations of AA. (D) Calibration curve of the generated H_2O_2 during the $CsPbBr_3$ catalyzing oxidation of different concentration of AA.

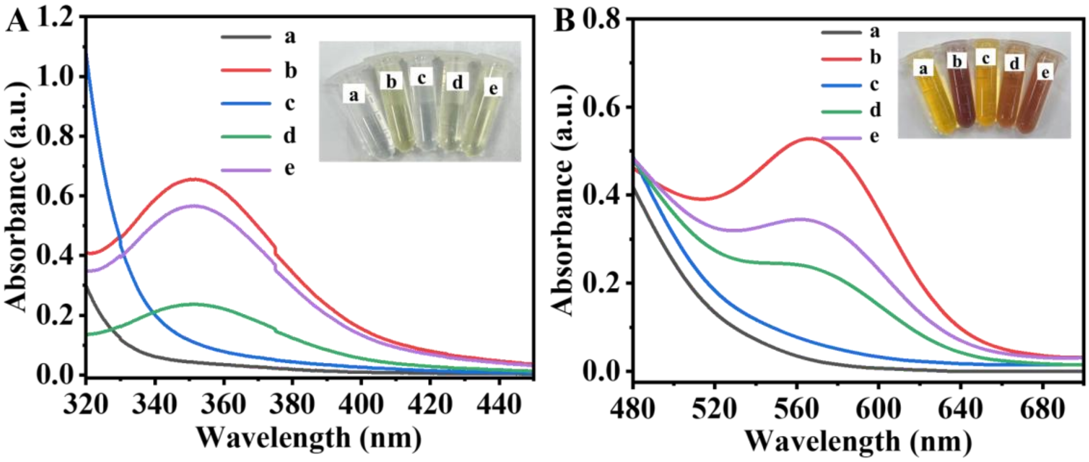

Figure S10. (A) Detection of H_2O_2 by iodide method. a: KI, b: KI+H_2O_2, c: KI+AA, d: KI+OPA-$CsPbBr_3$+AA (50 μM), e: KI+OPA-$CsPbBr_3$+AA (100 μM). (B) Detection of H_2O_2 by FOX assay. a: FOX, b: FOX+H_2O_2, c: FOX+AA, d: FOX+OPA-$CsPbBr_3$+AA (50 μM), e: FOX+OPA-$CsPbBr_3$+AA (100 μM).

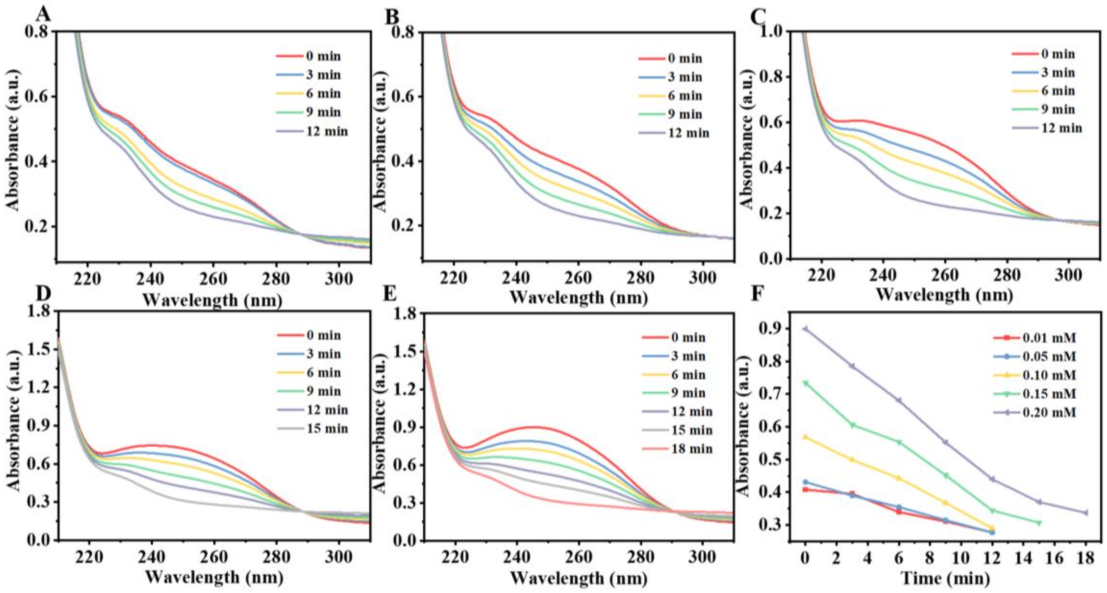

Figure S11. Real-time UV-vis absorption spectra of different concentrations of AA catalyzing oxidized by the OPA-$CsPbBr_3$ nanozyme. (A) 0.01 mM, (B) 0.05 mM, (C) 0.10 mM, (D) 0.15 mM, (E) 0.20 mM. (F) Time-dependent UV-vis absorbance at 259 nm of different concentrations of AA after catalyzing oxidized by OPA-$CsPbBr_3$ NCs.

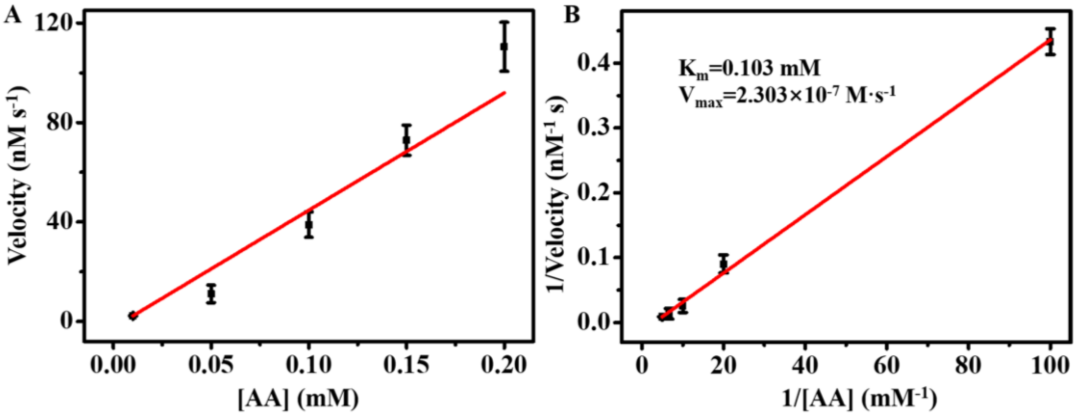

Figure S12. Steady-state kinetics of AA oxidation catalyzed by OPA-$CsPbBr_3$ NCs in HAc-NaAc buffer. (A) Velocity as a function of [AA]. (B) The Lineweaver-Burk plot.

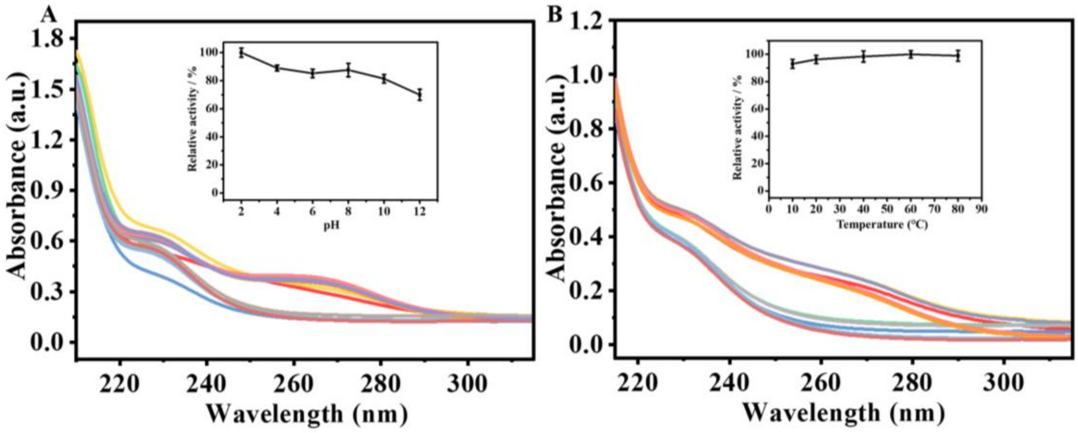

Figure S13. (A) UV-vis absorption curves of AA catalyzed by OPA-$CsPbBr_3$ NCs at different pH (inset shows relative activity). (B) UV-vis absorption curves of AA catalyzed by OPA-$CsPbBr_3$ NCs at different temperature (inset shows relative activity).

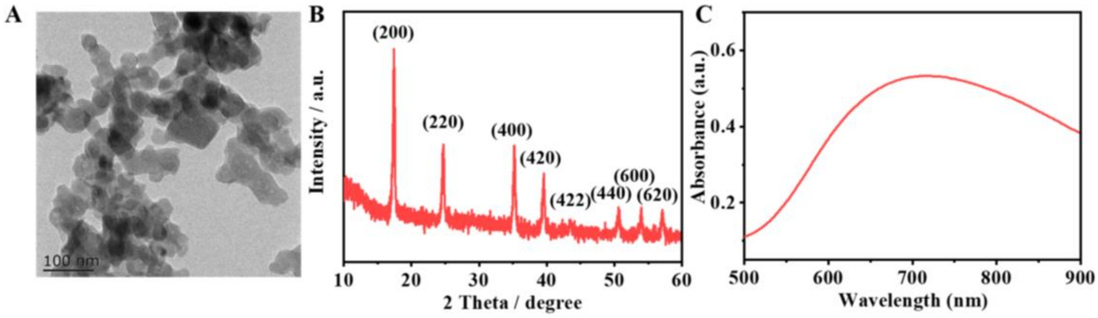

Figure S14. (A) TEM image, (B) XRD spectrum, (C) Absorption spectrum of PB nanoparticles.

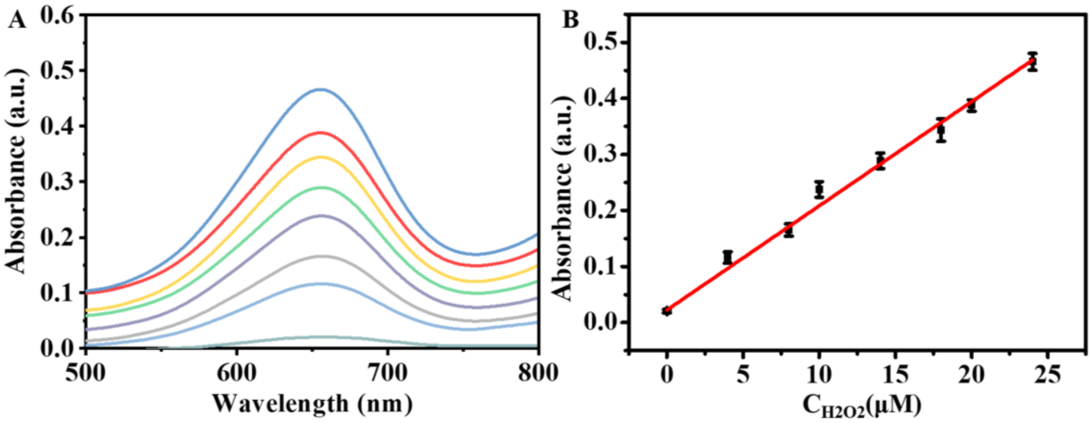

Figure S15. (A) UV-vis absorption spectra of PB-TMB-H_2O_2 system with different concentrations of H_2O_2. (B) Corresponding calibration curve.

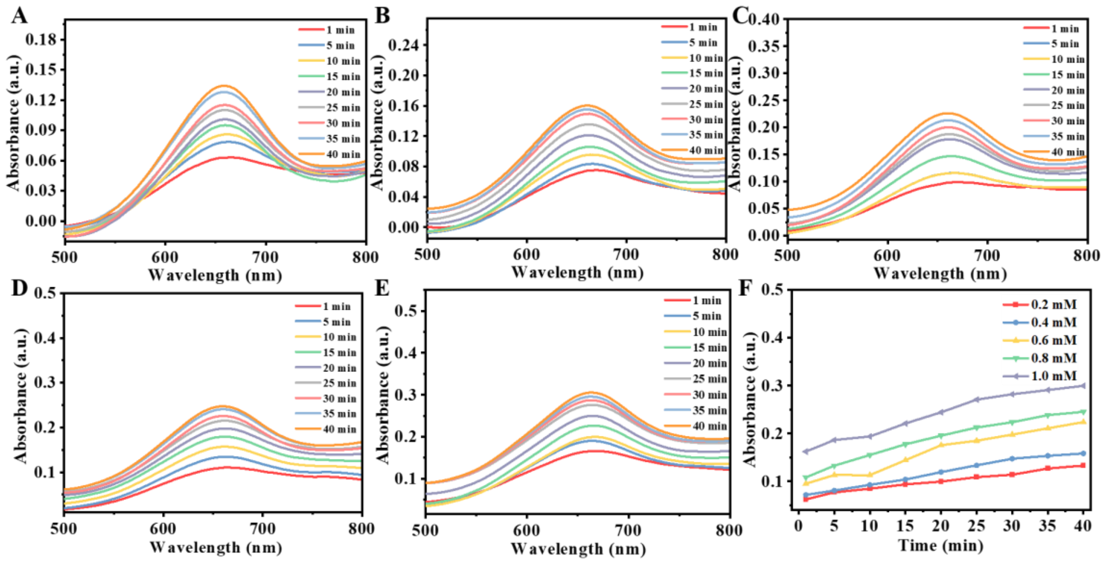

Figure S16. Real-time UV-vis absorption spectra of the PB catalyzed oxidation of different concentrations of TMB through the peroxidase-like activity. (A) 0.2 mM, (B) 0.4 mM, (C) 0.6 mM, (D) 0.8 mM, (E) 1 mM. (F) Time-dependent UV-vis absorbance at 652 nm with different concentrations of TMB.

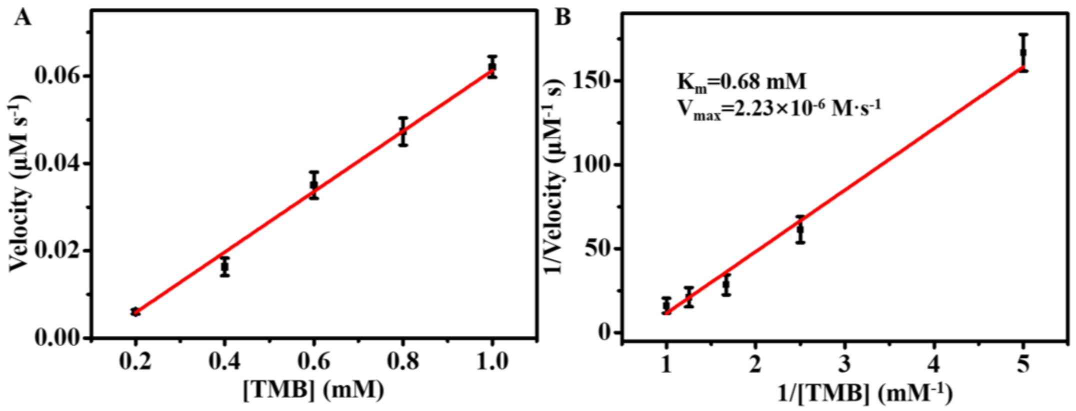

Figure S17. Steady-state kinetics for PB catalytic reaction with TMB and H_2O_2 (20 μM) in HAc-NaAc buffer. (A) The velocity as a function of [TMB]. (B) The Lineweaver-Burk plot.

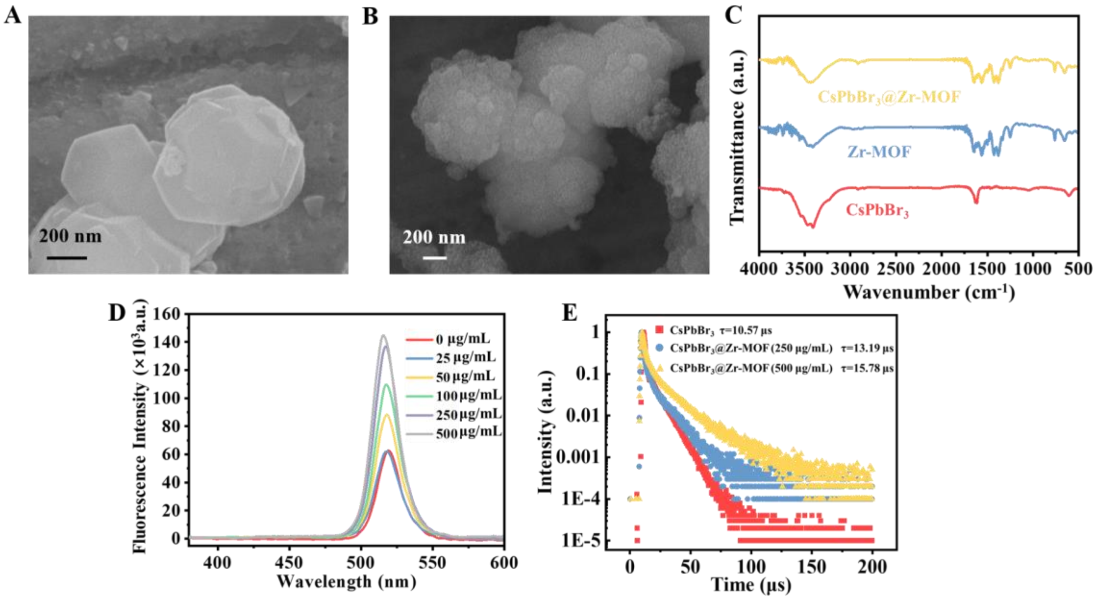

Figure S18. SEM images of (A) Zr-MOF and (B) $CsPbBr_3$@Zr-MOF nanocomposite. (C) FT-IR spectra of $CsPbBr_3$, Zr-MOF, and $CsPbBr_3$@Zr-MOF nanocomposite. (D) Fluorescence spectra of $CsPbBr_3$@Zr-MOF synthesized by adding different amounts of Zr-MOF. (E) Time-resolved FL decay curves of $CsPbBr_3$ at 520 nm for $CsPbBr_3$@Zr-MOF nanocomposite synthesized using different amounts of Zr-MOF.

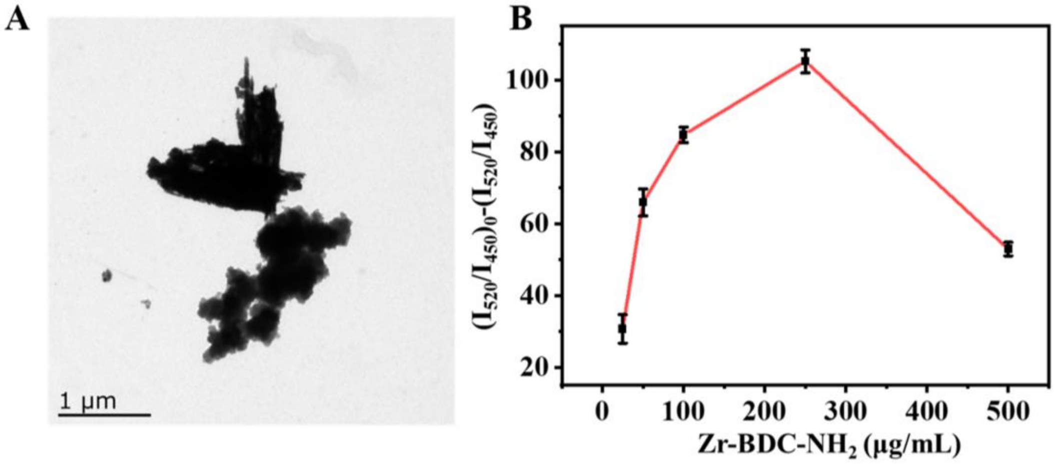

Figure S19. (A) TEM image of $CsPbBr_3$@Zr-MOF nanocomposite after addition of AA (500 μM). (B) The fluorescence intensity ratio variation of $CsPbBr_3$@Zr-MOF nanocomposite synthesized by adding different amounts of Zr-MOF before and after adding AA (0.1 mM).

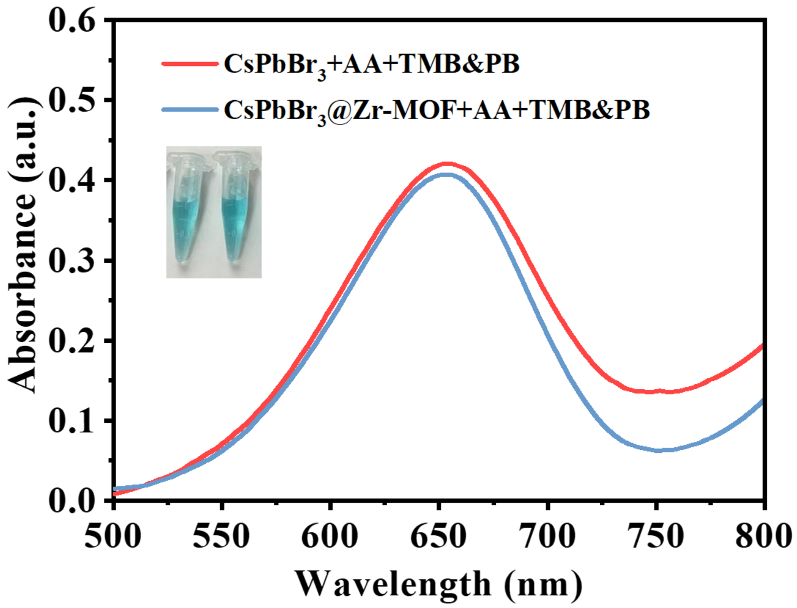

Figure S20. UV-vis absorption curves of the oxidation of TMB by AA catalyzed by $CsPbBr_3$ NCs and $CsPbBr_3$@Zr-MOF nanocomposite.

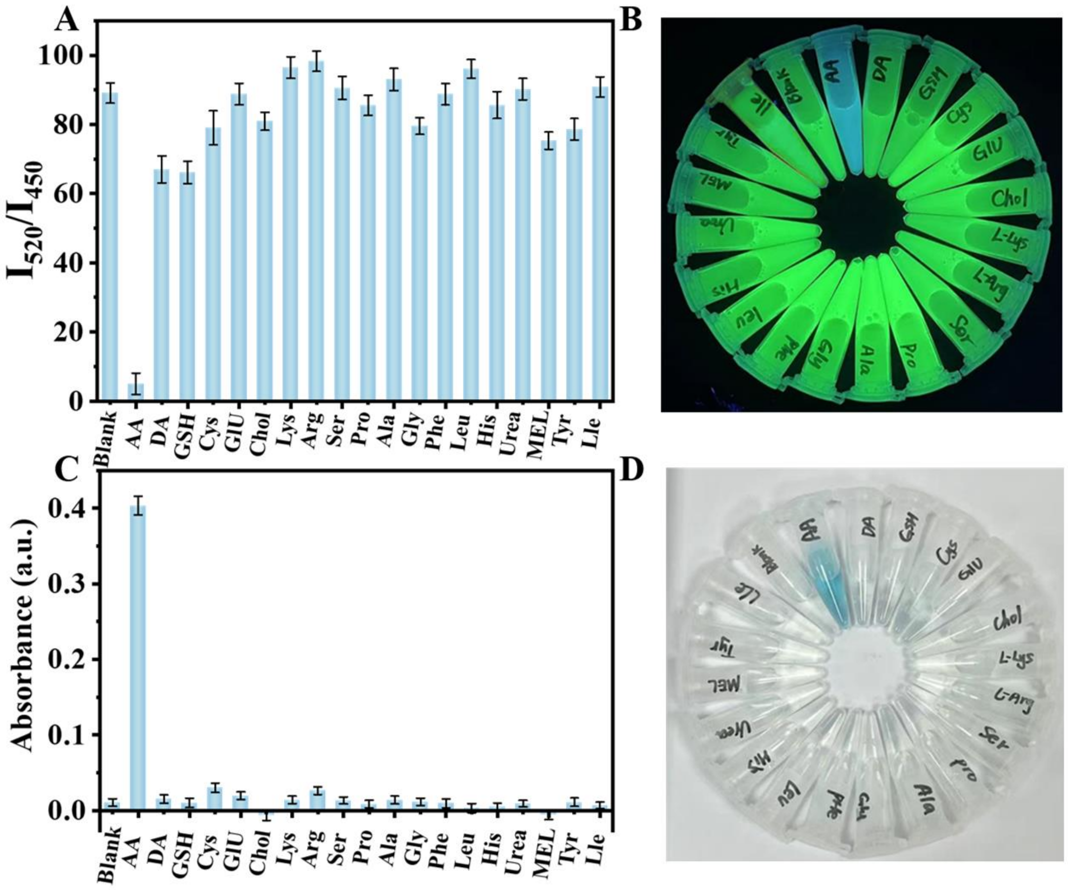

Figure S21. (A) The fluorescence intensity variation of $CsPbBr_3$@Zr-MOF nanocomposite in the presence of AA and other molecules (0.1 mM), respectively. (B) Image of the samples under 365 nm UV light irradiation. (C) The UV-vis absorbance of TMBox produced by the $CsPbBr_3$@Zr-MOF+PB nanozymes cascade reaction using AA and other molecules as the potential interference substrates. (D) Image of the samples.

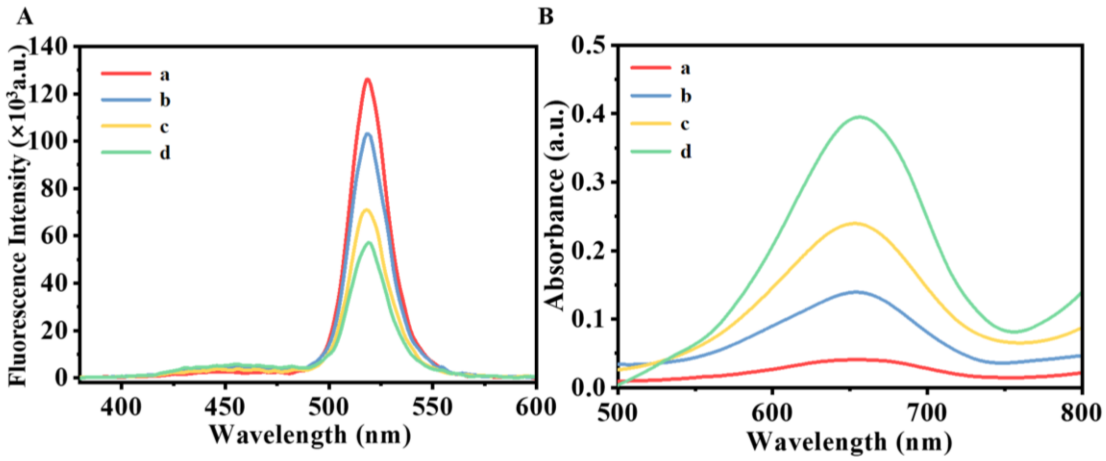

Figure S22. (A) Fluorescence spectra of $CsPbBr_3$@Zr-MOF after addition different concentrations of AA (a: 0 μM, b: 20 μM, c: 50 μM, d: 100 μM) in human serum sample. (B) UV-vis absorption spectra of TMBox produced by the $CsPbBr_3$@Zr-MOF+PB nanozymes cascade reaction system after adding different concentrations of AA (a: 0 μM, b: 20 μM, c: 50 μM, d: 100 μM) in human serum sample.

Table S1. Detailed results for the BET surface area, pore volume and pore size of OPA-$CsPbBr_3$ NCs.

	Surface area (m^2/g)	Pore volume (cm^3/g)	Pore size (nm)
OPA-$CsPbBr_3$	337.24	0.2835	2.3932

Table S2. Comparison of the K_m and V_{max} values of OPA-$CsPbBr_3$ for catalyzing substrate with other nature enzymes and mimetic oxidase.

Catalyst	Substrate	K_m (mM)	V_{max}	Ref.
Ascorbic acid oxidase	AA	0.050-0.064	23.70-26.82 μmol/min	[1]
Cholesterol oxidase	Chol	0.052	-	[2]
Glucose oxidase (GOx)	Glu	1.37	-	[3]
Glucose oxidase (GOx)	Glu	27		[4]
CeO_2	TMB	1.52	2.5×10^{-7} M·s^{-1}	[5]
Fe-N/C	TMB	0.62	5.26×10^{-7} M·s^{-1}	[6]
$Co_{0.6}$/NC-700	TMB	0.35	3.08×10^{-7} M·s^{-1}	[7]
CuFe-PBA-NC	TMB	0.09	25.62×10^{-6} M·s^{-1}	[8]
Heparin-OsNPs	TMB/Hg^{2+}	0.051	3.28×10^{-7} M·s^{-1}	[9]
Polydopamine-Fe^{3+} nanorod	TMB	0.095	4.37×10^{-8} M·s^{-1}	[10]
MOF-818	3,5-DTBC	0.81	3.17×10^{-6} M·s^{-1}	[11]
Mn/Fe-MIL(53)-2	TMB	0.34	-	[12]
CeO_2NPs-MOF	Cr(VI)	0.11	5.86×10^{-7} M·s^{-1}	[13]
OPA-$CsPbBr_3$ NCs	TMB	0.095	4.58×10^{-7} M·s^{-1}	This work

Table S3. Determination of AA in serum sample (dilute by 10-fold) by ratiometric fluorescent and colorimetric dual-mode sensor.

Sample	Detection method	Spiked (μM)	Found (μM)	Recovery (%)	RSD (%, n=3)
Human serum	Ratiometric fluorescence	0	2.42	0	0
		20	24.09	108.4	2.7
		50	50.79	96.7	2.1
		100	106.41	104.0	1.9
	Colorimetry	0	2.51	0	0
		20	23.79	106.4	1.7
		50	55.11	105.2	2.2
		100	101.84	99.3	2.6

S-References:

[1] S. Y. Leong, I. Oey, *Food Chem.* **2012**, *134*, 2075-2085.

[2] P. R. Solanki, A. Kaushik, A. A. Ansari, A. Tiwari, B. D. Malhotra, *Sens. Actuators B Chem.* **2009**, *137*, 727-735.

[3] J. Jung, S. Lim, *Appl. Surf. Sci.* **2013**, *265*, 24-29.

[4] A. Umar, M. M. Rahman, S. H. Kim, Y. B. Hahn, *J. Nanosci. Nanotechnol.* **2008**, *8*, 3216-3221.

[5] X. Cheng, L. Huang, X. Yang, A. A. Elzatahry, A. Alghamdi, Y. Deng, *J. Colloid Interface Sci.* **2019**, *535*, 425-435.

[6] Y. Wang, Z. Zhang, G. Jia, L. Zheng, J. Zhao, X. Cui, *Chem. Commun. (Camb.)* **2019**, *55*, 5271-5274.

[7] Y. Li, Y. Lu, X. Zhang, H. Cao, Y. Huang, *ACS Appl. Nano Mater.* **2021**, *4*, 9547-9556.

[8] R. P. Ojha, S. Pal, R. Prakash, *Microchem. J.* **2021**, *171*, 106854.

[9] S. B. He, Q. Q. Zhuang, L. Yang, M. Y. Lin, Y. Kuang, H. P. Peng, H. H. Deng, X. H. Xia, W. Chen, *Anal. Chem.* **2020**, *92*, 1635-1642.

[10] Y. Ai, H. Sun, Z. Gao, C. Wang, L. Guan, Y. Wang, Y. Wang, H. Zhang, Q. Liang, *Adv. Funct. Mater.* **2021**, *31*, 2103581.

[11] M. Li, J. Chen, W. Wu, Y. Fang, S. Dong, *J. Am. Chem. Soc.* **2020**, *142*, 15569-15574.

[12] L. Luo, Y. Ou, Y. Yang, G. Liu, Q. Liang, X. Ai, S. Yang, Y. Nian, L. Su, J. Wang, *J. Hazard. Mater.* **2022**, *423*, 127253.

[13] Y. Wang, R. P. Liang, J. D. Qiu, *Anal. Chem.* **2020**, *92*, 2339-2346.
